# Fibre orientation atlas guided rapid segmentation of white matter tracts

**DOI:** 10.1002/hbm.26578

**Published:** 2024-01-30

**Authors:** Fiona Young, Kristian Aquilina, Kiran K. Seunarine, Laura Mancini, Chris A. Clark, Jonathan D. Clayden

**Affiliations:** ^1^ Developmental Neurosciences Research and Teaching Department, UCL Great Ormond Street Institute of Child Health University College London London UK; ^2^ Department of Medical Physics and Biomedical Engineering University College London London UK; ^3^ Department of Neurosurgery Great Ormond Street Hospital for Children London UK; ^4^ Department of Radiology Great Ormond Street Hospital for Children London UK; ^5^ Lysholm Department of Neuroradiology, The National Hospital for Neurology and Neurosurgery University College London Hospitals NHS Foundation Trust London UK; ^6^ Department of Brain Repair and Rehabilitation, UCL Queen Square Institute of Neurology University College London London UK

**Keywords:** diffusion MRI, diffusion‐weighted MRI, fibre tractography, white matter, white matter tract segmentation

## Abstract

Fibre tract delineation from diffusion magnetic resonance imaging (MRI) is a valuable clinical tool for neurosurgical planning and navigation, as well as in research neuroimaging pipelines. Several popular methods are used for this task, each with different strengths and weaknesses making them more or less suited to different contexts. For neurosurgical imaging, priorities include ease of use, computational efficiency, robustness to pathology and ability to generalise to new tracts of interest. Many existing methods use streamline tractography, which may require expert neuroimaging operators for setting parameters and delineating anatomical regions of interest, or suffer from as a lack of generalisability to clinical scans involving deforming tumours and other pathologies. More recently, data‐driven approaches including deep‐learning segmentation models and streamline clustering methods have improved reproducibility and automation, although they can require large amounts of training data and/or computationally intensive image processing at the point of application. We describe an atlas‐based direct tract mapping technique called ‘tractfinder’, utilising tract‐specific location and orientation priors. Our aim was to develop a clinically practical method avoiding streamline tractography at the point of application while utilising prior anatomical knowledge derived from only 10–20 training samples. Requiring few training samples allows emphasis to be placed on producing high quality, neuro‐anatomically accurate training data, and enables rapid adaptation to new tracts of interest. Avoiding streamline tractography at the point of application reduces computational time, false positives and vulnerabilities to pathology such as tumour deformations or oedema. Carefully filtered training streamlines and track orientation distribution mapping are used to construct tract specific orientation and spatial probability atlases in standard space. Atlases are then transformed to target subject space using affine registration and compared with the subject's voxel‐wise fibre orientation distribution data using a mathematical measure of distribution overlap, resulting in a map of the tract's likely spatial distribution. This work includes extensive performance evaluation and comparison with benchmark techniques, including streamline tractography and the deep‐learning method TractSeg, in two publicly available healthy diffusion MRI datasets (from TractoInferno and the Human Connectome Project) in addition to a clinical dataset comprising paediatric and adult brain tumour scans. Tract segmentation results display high agreement with established techniques while requiring less than 3 min on average when applied to a new subject. Results also display higher robustness than compared methods when faced with clinical scans featuring brain tumours and resections. As well as describing and evaluating a novel proposed tract delineation technique, this work continues the discussion on the challenges surrounding the white matter segmentation task, including issues of anatomical definitions and the use of quantitative segmentation comparison metrics.

## Graphical abstract

A rapid atlas‐based direct white matter tract segmentation technique is extensively validated in three different datasets with consistent and strong results. Improved performance and explainability in the presence of pathology is demonstrated over alternatives methods.

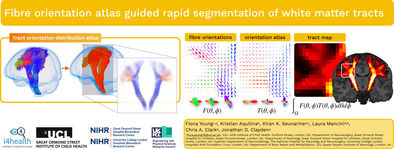

**Abbreviations**
AF: arcuate fasciculus
AT: atlas
CC: corpus callosum
CrP: cerebellar peduncles
CSD: constrained spherical deconvolution
CST: corticospinal tract
DKFZ: Deutsche Krebsforschungszentrum (German Cancer Research Center)
dMRI: diffusion magnetic resonance imaging
DSC: Dice similarity coefficient
DT: diffusion tensor
EC: external capsule
FACT: fibre assignment by continuous tracking
FOD: fibre orientation distribution
HARDI: high angular resolution diffusion imaging
HCP: human connectome project
IFOF: inferior fronto‐occipital fasciculus
MNI: Montreal Neurological Institute
MRI: magnetic resonance imaging
MSMT: multi‐shell multi‐tissue
OR: optic radiation
ROI: region of interest
SH: spherical harmonic
SFOF: superior fronto‐occipital fasciculus
SLF: superior longitudinal fasciculus
SSST: single‐shell single‐tissue
TF: tractfinder
TG: tractography
TGR: reference tractography
TOD: track orientation distribution
TS: TractSeg

## 1 INTRODUCTION

Diffusion weighted magnetic resonance imaging (dMRI), with its sensitivity to microstructural features not detectable by other non‐invasive means, continues to bring vast benefits to neuroimaging for both clinical and research applications. In high angular resolution diffusion imaging (HARDI), the acquisition of a large number (≳40) of volumes with different diffusion weighted gradient directions enables the detailed probing of diffusion anisotropy in brain tissue and, by extension, of orientational organisation of tissue microstructure. HARDI is an ideal tool for mapping brain white matter, which consists of tightly packed neuronal fibres connecting different brain regions and exhibits strong diffusion anisotropy along the fibre orientation directions. White matter fibres are arranged in a complex configuration of distinct bundles, or tracts, associated with specific neurological or cognitive functions. The delineation of these tracts is a key processing step for many neuroscientific and clinical imaging pipelines, be it for the studying of effects in specific pathways, modelling the brain's structural ‘connectome’, or for the identification of pathways at risk of injury from neurosurgical procedures.

Streamline tractography remains the most widely adopted tool for reconstructing white matter tracts from dMRI data in both clinical and research settings. The technique involves seeding individual ‘streamlines’ within the brain which are then propagated stepwise along directions determined by local fibre orientations as determined from the diffusion data. While that simple process of seeding and tracking underpins all tractography, actual implementations branch into an extensive taxonomy utilising a range of fibre orientation models and representations, tracking algorithms, termination criteria and seeding strategies. This is discussed extensively in existing literature (Essayed et al., 2017; Jeurissen et al., 2019; Maier‐Hein et al., 2017; Rheault, Poulin, et al., 2020; Yamada et al., 2009; Yang et al., 2021). To achieve individual tract segmentations, tractography streamlines are assigned to tracts using variety of approaches including selection based on logical regions of interest (ROIs) and clustering, discussed in more detail below. We will use the term ‘streamline tractography’ to refer to the computational methodology itself and to the derived specific tract reconstructions and segmentations.

In neurosurgical settings, tractography is frequently based on diffusion tensor (DT) fibre orientation models (Toescu et al., 2020; Yang et al., 2021) and deterministic tracking algorithms, for example as implemented in Brainlab's iPlan® (single tensor) (Brainlab, 2012) and Elements (dual tensor) (Sollmann et al., 2020) Fibre tracking applications (Brainlab AG, Munich, Germany) which use a version of fibre assignment by continuous tracking, or FACT (Mori et al., 1999). In research settings, diffusion tensor models and deterministic algorithms have long since given way to multi‐fibre models and probabilistic algorithms. These include multi‐tensor models (Peled et al., 2006), Q‐ball imaging (Tuch, 2004), ball‐and‐sticks (Behrens et al., 2003) and constrained spherical deconvolution (CSD)‐derived fibre orientation distribution functions on the modelling side, and probtrackx (Behrens et al., 2007), first or second‐order integration over FODs (iFOD1/iFOD2) (Tournier et al., 2010) and particle filter tractography (Girard et al., 2014) on the algorithms side.

Regardless of the particular combination of fibre model, algorithm and tracking criteria, streamline tractography is compromised by weaknesses that can lead to flawed results or interpretations if not accounted for (Rheault, Poulin, et al., 2020; Schilling et al., 2019; Schilling et al., 2022). Although probabilistic tractography is generally considered to be superior to deterministic tractography in its ability to reconstruct bundles more completely, especially ones with complex shapes, sharp bends and fanning, this improved sensitivity is accompanied by a high propensity for false positive streamlines (Maier‐Hein et al., 2017). As a result, targeted reconstruction of specific bundles using probabilistic tractography requires constraining with inclusion and exclusion regions of interest (ROIs), whose manual placement is labour intensive and demands expert neuroanatomical knowledge.

Nonetheless, there is growing consensus that (pending appropriate regulatory approval) the clinical community ought to adopt probabilistic, non‐DT tractography (Beare et al., 2022; Petersen et al., 2017; Yang et al., 2021), given, among other issues, the low sensitivity of DT deterministic tractography particularly around lesions (Ashmore et al., 2020). There is evidence that a shift is underway, at least in the context of presurgical planning (Toescu et al., 2020), although there is the question of whether the adoption of advanced tractography is being driven from the supply or demand side. Until recently, commercially available neurosurgical navigation platforms have exclusively supported diffusion tensor modelling and deterministic tractography (a recent exception is the Medtronic Stealth™ Tractography application [Medtronic, USA], which implements CSD‐based tractography; Pozzilli et al., 2023, as well as DT). This lack of readily available alternatives in the neurosurgeon's workflow and certified for safe clinical use is undoubtedly a major factor in the persisting preference for deterministic methods in clinical practice. But perhaps demand for probabilistic tractography in neuronavigational software has also been understandably muted on account of its lower ease of use and practicality. DT acquisitions can have as few as six diffusion weighted directions, resulting in much shorter scan times compared to full HARDI acquisitions. Deterministic tracking itself is rapid, and the placement of ROI need not be as strict as with probabilistic tractography owing to a lower sensitivity to false positives (O'Donnell et al., 2017). A lack of availability of the necessary expertise and time limits neurosurgical centres' access to state‐of‐the‐art tractography (Toescu et al., 2020). There is thus an unmet need for reliable, automated tract segmentation techniques that can rapidly provide consistent and complete tract reconstructions for clinical use.

### 1.1 Streamline‐based automatic tract segmentation

A key issue with streamline tractography is it is lack of reproducibility: A combination of frequent false positives, variations in anatomical tract definitions, and individual preferences and variations in ROI placement lead to huge differences in reconstructions of the same tract across operators, making it difficult to compare and reuse results (Schilling et al., 2021). Automating the placement of anatomical constraints or transfer of anatomical knowledge could improve tractography's reproducibility. Several automated tractography approaches to bundle segmentation have been proposed, which broadly comprise atlas‐based automatic ROI placement and streamline clustering approaches. TRACULA (Yendiki et al., 2011) and TractQuerier (Wassermann et al., 2016) are examples of the former which rely on FreeSurfer parcellations to apply anatomical priors to tractography, while XTRACT (Warrington et al., 2020) utilises a set of predefined ROIs to be registered to the subject. The additional step of either comprehensive and robust tissue parcellation, typically using computationally intensive tools such as FreeSurfer, or accurate registration, unfortunately limits the accessibility of these approaches to clinical and even (to a lesser extent) research applications.

RecoBundles (Garyfallidis et al., 2018), white matter analysis (O'Donnell et al., 2017; O'Donnell & Westin, 2007), atlas based adaptive clustering (Tunç et al., 2014), example‐based automatic tract labelling (Yoo et al., 2015) are all examples of data driven, group‐wise streamline clustering and matching approaches. Another approach, named Classifyber, uses linear classification of streamline features to label streamlines belonging to the target bundle in a new subject in around 3 min per bundle (Bertò et al., 2021). In all clustering approaches, the necessary generation of whole brain tractograms and in some cases the additional construction of example or reference tractography data, as well as, in some cases, long processing times and high memory requirements present barriers to application. The above techniques also ultimately still require streamline tractography at the point of application in a new subject, and are thus not free of its biases and inaccuracies already referenced. There have also been recent developments in the application of machine learning to tractography (Poulin et al., 2019), a promising field that is still in its infancy and a while off from widespread adoption and clinical translation.

### 1.2 Streamline free white matter segmentation

A further set of prior works address the problem of tract segmentation while forgoing the use of streamline tractography entirely. These include deep learning models for direct segmentation from fibre orientation representations (Li et al., 2020), a multi‐label supervised clustering approach (Ratnarajah & Qiu, 2014) and level‐set and front propagation situations (Hao et al., 2014; Nazem‐Zadeh et al., 2011). Deep learning‐based approaches have the advantage of producing highly reproducible results in short processing time, without, in the case of TractSeg, the need for template registration. However, drawbacks of direct, deep learning‐based methods which produce binary segmentations include a lack of explainability, and a dependence on large volumes of annotated training data which are labour‐intensive to produce. This limits their flexibility: if a user requires a tract segmentation which is either anatomically different or not covered by an existing pre‐trained model, then the necessary production of new training data and subsequent model training represents a high logistical and computational barrier. There is also the question of robustness in clinical data, particularly those featuring gross structural pathologies such as tumours. In addition to healthy data, Neuro4Neuro (Li et al., 2020) was validated only in a dementia dataset, and TractSeg was qualitatively validated in schizophrenia and autism datasets in the original work. TractSeg has also been qualitatively validated in a tumour dataset with mostly successful results, with more complete segmentations in cases with minimally displacing tumours (Richards et al., 2021). In Moshe et al. (2022), the authors trained their own TractSeg model, on approximately 500 datasets, to segment the corticospinal tract (CST) in brain tumour patients. The results were more reproducible than for the compared manual method, and obtained an average dice similarity score of 0.64, almost 25% worse than the performance in healthy data reported in the original TractSeg study (for the same tract). The authors cite a lack of reliable and sufficient labelled training data as a reason for limiting their study to a single tract, despite the importance of other tracts in preoperative fibre mapping.

Our aim is to develop a streamline free tract segmentation technique incorporating spatial and orientational priors, with a focus on computational simplicity, specificity, and intuitive output. Briefly, the proposed method consists of a tract‐specific orientation distribution atlas, which encodes spatial and orientational prior information about each tract, which is then directly compared with the diffusion data derived fibre orientation distribution information in the target image via an inner product operation on the two spherical distributions. Here we report on extensive validation of our approach applied to the CST, optic radiation (OR), arcuate fasciculus (AF) and inferior fronto‐occipital fasciculus (IFOF). Those four tracts were chosen due to their high clinical relevance and frequent involvement with surgical targets: In Toescu et al. (2020) they were cited by neurosurgeons as the most frequently reconstructed tracts, followed by the corpus callosum. Generalisation to additional tracts is straightforward and requires relatively few training subjects, which is ideal in clinical translation, where appropriate data is often hard to obtain. Furthermore, the anatomical definitions of many tracts are not universally agreed upon, and new or understudied tracts can gain new neurosurgical importance, making it all the more important that methods can be retrained or generalised to new applications using a small number of samples. Preliminary results for this approach have been published in Young et al. (2022), where we focused on qualitative results in the context of neurosurgical subjects with significantly deforming tumours. Here we provide a more detailed description of the technique and two additional tract atlases, as well as extensive quantitative validation of general applicability to both healthy and clinical datasets.

Our work bears much similarity of intuition and approach to that in Bazin et al. (2011) (‘Diffusion‐Oriented Tract Segmentation’ or DOTS), but with several practical differences. DOTS is based on diffusion tensor modelling, with the atlas' direction prior consisting of a single principal direction per voxel, rather than a full spherical distribution as in our approach. The comparison between the atlas and subject data to be segmented consists of several mathematical steps involving Markov random field models, neighbouring tensor connectivity, and propagation of probabilities, compared to our approach of taking the inner product of two spherical distributions. The diffusion tensor atlas described in Hagler et al. (2009) also proposes a similar concept. There, a fibre location and orientation atlas is created by averaging the diffusion tensor and tractography‐derived information from multiple subjects and subsequently used to estimate the voxel‐wise a posteriori tract probability in a single subject. In contrast to our proposed atlas, orientation information was encoded by averaging diffusion tensor principal eigenvectors across subjects, instead of a tract‐specific approach to orientation as in our case. And in Hagler et al. (2009), spatial probability is given by the averaged, normalised track density values from individual deterministic streamline tractography, even though equating streamline density with likelihood of tract location is problematic (Rheault et al., 2019; Smith et al., 2013). Finally, there are similarities with the bundle specific tractography approach described in Rheault et al. (2019), in particular the use of a streamline‐template and track orientation distribution (TOD) mapping (Dhollander et al., 2014), and extension of track density imaging (Calamante et al., 2010) into the angular domain, to incorporate orientational priors. However, the purpose of the TOD prior in that work was to enhance tractography, whereas our aim is to forgo tractography entirely at the point of application in a new subject.

### 1.3 Terminology

Throughout this paper, we adopt the terminology set out in Côté et al. (2013). In particular we will mainly use the term *tract* (synonymous with *fibre bundle* in Côté et al., 2013) to refer to an anatomical structure of a group of fibres with a common functional or anatomical organisation. Individual tracts are the targets of white matter segmentation. The term *bundle* (shortened from *streamline bundle*) will be used to refer to a collection of tractography *streamlines*, usually serving as a representation of, but not synonymous with, a corresponding anatomical tract. In some contexts, to maintain consistency with prior publications, the term *track* may be used in place of streamline.

## 2 METHODS

The proposed technique will be described in two stages. The first involves the definition and construction of tract‐specific orientation atlases, which are created once for all subsequent applications. Secondly, the atlas is used in combination with a dMRI derived fibre orientation distribution (FOD) to segment the tract in a new subject.

### 2.1 Atlas construction

The key component of the proposed tract mapping method is the use of a tract‐specific atlas of fibre bundle orientation and location. The purpose of the tract atlas is to capture and store prior anatomical knowledge of a given tract, including its typical location and orientation across subjects. While this is hereinafter referred to simply as a tract orientation atlas, and this section will focus on the orientation component, each tract atlas incorporates both orientational and spatial information.

**FIGURE 1:**
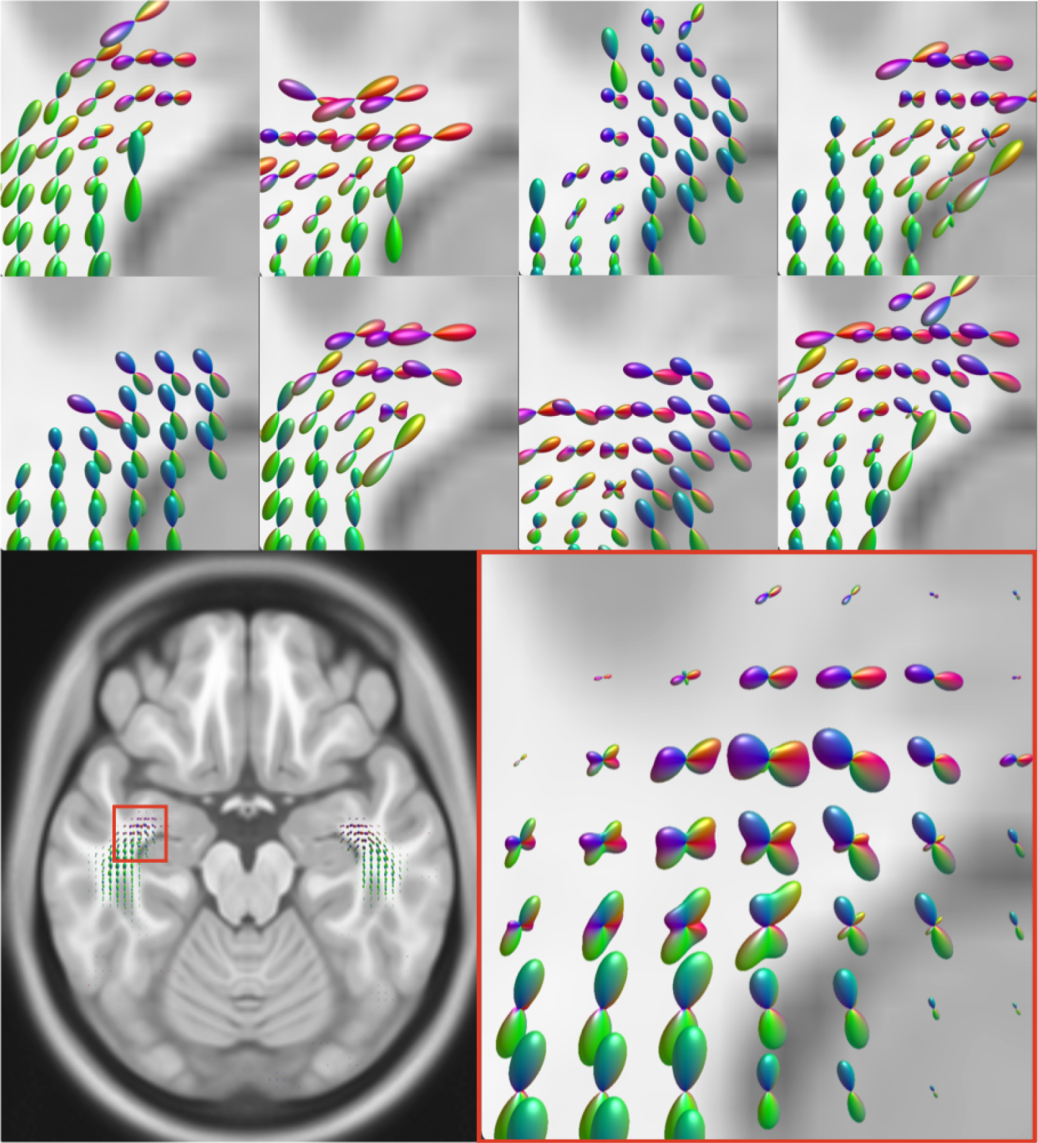
Averaging of individual normalised TOD maps for tract atlas. At the anterior portion of Meyer's loop (indicated with red box in axial MNI slice), there is high inter‐subject variability in streamline orientations (top two rows, random subset of training subjects shown). When averaged, that orientational variability is captured as broader distributions, with more frequently visited voxels obtaining higher overall amplitude.

The objective is to create a map in a template space capturing, at each location, the range of possible orientations the tract can take on as a single spherical distribution. A narrow distribution may be found where the tract's orientation is highly consistent across all subjects, whereas a more spread‐out distribution would reflect a wider range of possible orientations, which may be seen in regions of fanning or sharp turning (Figure 1).

To obtain such a mapping, a combination of streamline tractography and TOD mapping (Dhollander et al., 2014) is used. While tractography has significant limitations as discussed above, it remains the standard way of segmenting white matter bundles from in vivo dMRI data, and biases and errors can, with appropriate post‐processing steps, be at least partially corrected for. In addition, tractography uniquely enables the extraction of orientation information specific to the reconstructed bundle, which would not be possible from a binary voxel‐wise segmentation.

A dataset of 16 healthy adult HARDI acquisitions (‘EEG, fMRI and NODDI dataset’; Clayden & Deligianni, 2020, available online at osf.io/94c5t) was used in the creation of reference bundles for developing the atlas. We have shown that this number of subjects is sufficient for capturing inter‐subject variability, and that increasing the number of training subjects does not improve performance (see Young et al., 2023). In each subject, the bundle of interest was reconstructed in both hemispheres using probabilistic streamline tractography with iFOD2 (Tournier et al., 2010) and a consistent ROI strategy based on anatomical landmarks broadly agreed upon in prior works. (See Appendix D for full details on tractography parameters and ROI strategies used for each tract.)

**FIGURE 2:**
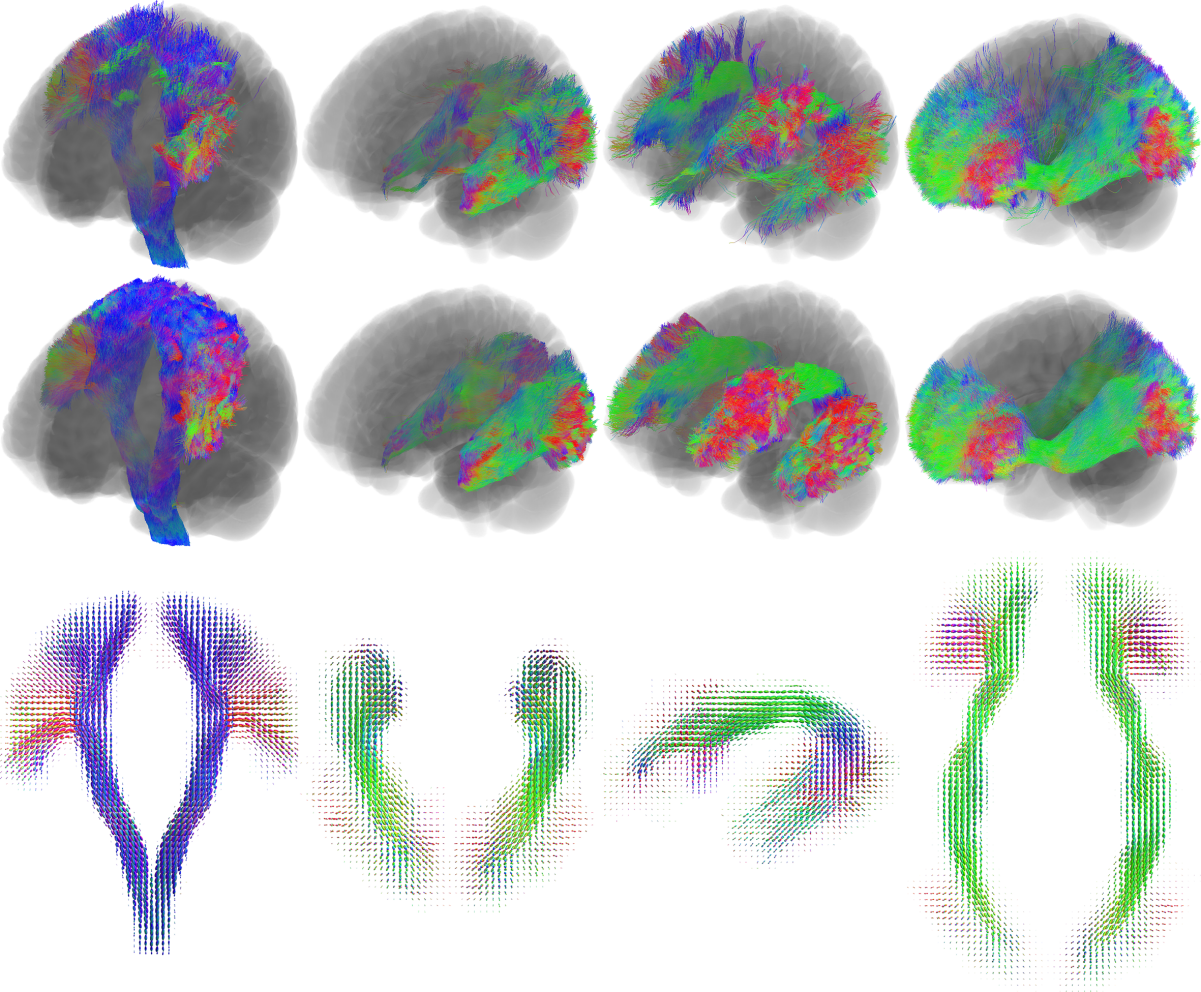
Atlas construction data for (from left to right) the corticospinal tract (CST), optic radiations (OR), arcuate fasciculus (AF) and inferior fronto‐occipital fasciculus (IFOF). Top: streamlines removed via manual filtering. Middle: filtered streamline bundles (all subjects combined). Bottom: tract orientation atlases, composite images in coronal (CST), axial (OR, IFOF) and sagittal (AF) views. All figures depicted in MNI152 reference space.

After streamline generation, each streamline bundle was transformed into MNI space using affine registration implemented in FMRIB's Linear Image Registration Tool (Jenkinson et al., 2002) between the subject's T1‐weighted image and the MNI152 T1 template (Fonov et al., 2011). Affine registration rather than non‐linear, was used for this step to capture individual anatomical variation and minimise unrealistic warping of streamlines from local registration errors or overfitting. With all subject streamlines aggregated in MNI space, manual filtering of streamlines was performed (Figure 2) to remove not only ‘volumetric false positives’, which depart from the accepted volume of the tract, but also ‘orientational false positives’ (OFPs), which remain entirely within the tract volume but are at least in part aligned with a different, intersecting bundle. An example of such OFPs is depicted in Supplementary Figure E.9. Such streamlines have little effect on any volumetric applications of the reconstruction, e.g. via a track density depiction. However, their removal is vital for the construction of the orientation atlas, which summarises the orientational distribution of streamlines on a voxel‐wise basis. Filtering was performed in DSI studio (v2021_04, https://dsi-studio.labsolver.org/) (Yeh, 2023), which enables the filtering of streamlines based on angle of intersection with a cutting plane. The percentage of streamlines filtered for each tract and summarised reasons for removal are presented in Table 1.

**TABLE 1:** Streamline filtering statistics.

		Original	Filtered	Difference	Reduction (%)	Reasons for discarding
CST	Left	148,833	145,300	3533	2.37	Contamination from: AF/SLF, SFOF, CC, CrP
	Right	144,759	139,019	5740	3.97	
	Total	293,592	284,319	9273	3.16	
AF	Left	61,922	49,778	12,144	19.61	Contamination from: EC, CST, CC Overextension into: Motor, anterior temporal, and superior frontal cortex
	Right	61,834	43,027	18,807	30.42	
	Total	123,756	92,805	30,951	25.01	
OR	Left	123,842	99,984	23,858	19.26	Contamination from: Tapetum of CC, SLF
	Right	122,534	109,265	13,269	10.83	
	Total	246,376	209,249	37,127	15.07	
IFOF	Left	80,000	44,224	35,776	44.72	Contamination from: Tapetum of CC, VOF, superior frontal cortex
	Right	80,000	31,753	48,247	60.31	
	Total	160,000	75,977	84,023	52.51	

Abbreviations: AF, arcuate fasciculus; CC, corpus callosum; CrP, cerebellar peduncles; CST, corticospinal tract; EC, external capsule; IFOF, inferior fronto‐occipital fasciculus; SFOF, superior fronto‐occipital fasciculus; SLF, superior longitudinal fasciculus.

After aggregate filtering, the retained streamlines were re‐separated into individual subject bundles and the TOD was computed from the individual bundles as described in Dhollander et al. (2014) and implemented in MRtrix3 (Tournier et al., 2019). TOD mapping is the generalisation of track density imaging into the angular domain, creating a 5D spatio‐angular representation of streamline tracks on a voxel‐wise basis. The TOD image is represented in modified spherical harmonic basis (Descoteaux et al., 2006) using only even orders up to a maximum order $l_{\max}=8$, meaning each image consists of 45 coefficients, denoted $t_{j}$, per voxel. The distribution is described by those coefficients and the modified spherical harmonic basis functions $Y_{l,m}$ (Descoteaux et al., 2006) as

(1) $$T\left(\theta ,\phi \right)=\sum_{l=0}^{l_{\max}}\sum_{m=-l}^{l}t_{l,m}Y_{l,m}\left(\theta ,\phi \right)=\sum_{j}t_{j}Y_{j}\left(\theta ,\phi \right).$$

The individual TOD images at this stage still contain significant density bias, with exaggerated differences in magnitude between the core bundle portions and fanning extremities owing to tractography's tendency towards early termination outside of the densest collinear tract regions (Rheault, Poulin, et al., 2020; Smith et al., 2013). The purpose of the atlas is to capture only the likelihood of a tract's presence in any given voxel (spatial prior) and, in the case that it is present, its expected orientation (orientational prior). If the spatial prior is to be determined by considering the spatial variation of the tract between subjects, then the only information needed for each individual subject is a binary visitation map for the bundle and orientational data. Thus to remove the streamline density component, the TOD maps for each subject are normalised as follows. The spherical integral of each SH basis function $Y_{l,m}$ is

(2) $$\int_{\Omega }Y_{l}^{m}\left(\theta ,\phi \right)\;d\Omega =\left\{\begin{array}{cc} \sqrt{4\pi }\; & \;\text{if}\;l=m=0\\ 0\; & \;\text{otherwise}\; \end{array},\right.$$

where $\int_{\Omega }d\Omega$ is the spherical integral over the whole 2‐Sphere $S^{2}$. Using the sum and constant rules of integration, the spherical integral of $T\left(\theta ,\phi \right)$ is

(3) $$\int_{\Omega }T\left(\theta ,\phi \right)\;d\Omega =t_{0}\sqrt{4\pi },$$

where $t_{0}$ is the first SH coefficient for l=m=0. Thus to remove density information the TOD map is normalised to unit integral as

(4) $$\tilde{T}\left(\theta ,\phi \right)=\frac{T\left(\theta ,\phi \right)}{\sqrt{4\pi }t_{0}}$$

After each individual TOD map has been normalised in MNI space, what remains contains only information about the tract's streamline orientations, and none about the number of streamlines passing through a given voxel in the original reconstruction.

Finally, the mean of all individual normalised TOD maps is computed to produce the final population tract TOD atlas. Averaging all maps results in distributions that reflect all possible ranges of tract orientations in each voxel (Figure 1), while the first SH coefficient of the atlas will reflect the proportion of training subjects in which the tract was present in a given voxel. Outlier voxels visited by streamlines in only a single subject's reconstruction will contribute little weight to the final atlas. This atlas can then be registered to a target subject for further processing. Atlases have so far been created for the most commonly indicated pathways in neurosurgical planning and guidance, namely the corticospinal tract (CST), optic radiations (OR), arcuate fasciculus (AF) and inferior fronto‐occipital fasciculus (IFOF; Figure 2), with the creation of further atlases to be the subject of future work. Users can generate custom atlases from a set of training streamlines using the code available at https://github.com/fionaEyoung/tractfinder. Tract orientation atlases will be made available prior to publication, pending approval from the assignees of the relevant IP.

### 2.2 Inner product

The orientation atlas is registered from MNI to subject space using affine registration of the structural images, e.g. T1 weighted scans (here we again use FMRIB's Linear Image Registration Tool; Jenkinson et al., 2002, although other registration tools are equally suitable; Visser et al., 2020). Using affine registration ensures that the technique remains fast and robust at the point of application, and experiments have indicated that non‐linear atlas registration offers no advantage in accuracy (see Supplementary Material A). The tract atlas intentionally conveys a degree of spatial tolerance to account for individual variations in tract location, with the following step acting to refine the estimate according to observed local information in the target image. The objective is to obtain a measure per voxel of how closely the predicted tract orientation distribution overlaps with the observed FOD, modelled from dMRI data using constrained spherical deconvolution (CSD) (Tournier et al., 2007).

This can be achieved by taking the inner product of the two functions, that is, multiplying them and integrating the product over all spherical angles. As with the TOD atlas, the FOD is represented in the modified spherical harmonic (SH) basis:

(5) $$F\left(\theta ,\phi \right)=\sum_{l=0}^{l_{\max}}\sum_{m=-l}^{l}f_{l,m}Y_{l,m}\left(\theta ,\phi \right)=\sum_{j}f_{j}Y_{j}\left(\theta ,\phi \right).$$

The spherical integral of the product of two spherical harmonic basis functions $Y_{l_{1},m_{1}}$ and $Y_{l_{2},m_{2}}$ is

(6) $$\int_{0}^{\pi }\int_{0}^{2\pi }Y_{l_{1},m_{1}}\left(\theta ,\phi \right)Y_{l_{2},m_{2}}\left(\theta ,\phi \right)\sin\left(\theta \right)\text{dθdϕ}$$

(7) $$=\delta_{m_{1},m_{2}}\delta_{l_{1},l_{2}},$$

where $\delta_{i,j}$ is the Kronecker delta defined as $\delta_{i,j}=1\;\text{if}\;i=j\;\text{else}\;0$. Therefore, for two functions $F\left(\theta ,\phi \right)$ and $T\left(\theta ,\phi \right)$ the integral of their product can be expressed as

(8) $$\begin{array}{l} \int_{0}^{\pi }\int_{0}^{2\pi }F\left(\theta ,\phi \right)T\left(\theta ,\phi \right)\sin\left(\theta \right)\text{dθdϕ}\\ =\int_{0}^{\pi }\int_{0}^{2\pi }\left(\sum_{j}f_{j}Y_{j}\left(\theta ,\phi \right)\right)\left(\sum_{k}t_{k}Y_{k}\left(\theta ,\phi \right)\right)\sin\left(\theta \right)\text{dθdϕ}\\ =\sum_{k,j}f_{j}t_{k}\delta_{\mathrm{jk}}. \end{array}$$

Thus for two distributions represented by a vector containing their spherical harmonic coefficients, the integrated product can be obtained by taking the inner product of the two coefficient vectors. The final result is this voxel‐wise inner product of the registered atlas and subject FOD images. The resulting image is a pseudo‐probability map of tract location, in arbitrary and dimensionless units. Typical values range from [0 to 0.5], with 0.05 empirically determined to be a suitable threshold for converting to binary segmentation. We refer to this proposed segmentation approach, of registering a pre‐constructed orientation atlas to a target image and computing the inner product as described as ‘tractfinder’. Tractfinder is available as an external MRtrix3 module at https://github.com/fionaEyoung/tractfinder. The method requires a tract orientation atlas in standard space, the FOD image of a new subject from which to segment the tract, and structural scans (e.g. T1‐weighted) in standard and subject space to perform registration. Using these inputs, tractfinder is fully automated, although the user can specify a custom value for thresholding. An addition tumour deformation modelling step is also possible (not covered here; see Young et al., 2022), which requires a segmentation of a tumour in subject space.

### 2.3 Quantitative evaluation

While a ground truth for white matter tract segmentation is not obtainable in vivo, we compare our technique with two other widely adopted methods for a quantitative estimation of reliability and accuracy. Results are presented for three different datasets: Two large healthy datasets and one smaller dataset of clinical neurosurgical acquisitions, together covering a range of acquisition protocols and scanner specifications. In segmentation tasks, it is common to present a single numeric score of similarity with a ground truth by way of establishing accuracy. However, in the absence of a ground truth for this particular task, we aim to present as rounded a picture as possible of the differences and characteristic features of each method through a range of different volumetric distance‐based similarity metrics. The purpose of this validation is therefore not to determine which method is best, as indeed cannot be determined without a reliable reference point, but to highlight the ways in which they are similar, and their characteristic tendencies.

#### 2.3.1 Data

We considered three different datasets with which to compare the proposed method against alternative tract segmentation methods. Each dataset and any dataset‐specific preprocessing is described below. In addition, for all subjects we performed brain masking (Tournier et al., 2019), linear registration (Jenkinson et al., 2002; Jenkinson & Smith, 2001) (FSL v.6.0 Linear Image Registration Tool) between subject space and MNI152 (Fonov et al., 2011) space and two versions of constrained spherical deconvolution (CSD): single‐shell, single‐tissue (SSST) CSD (‘original flavour’) (Tournier et al., 2007; Tournier et al., 2019) and multi‐shell, multi‐tissue CSD (Jeurissen et al., 2014) restricted to white matter and grey matter tissue compartments. In both cases, response functions were obtained using the Dhollander unsupervised 3‐tissue response function estimation algorithm (Dhollander et al., 2016, 2019). All CSD processing was conducted using the MRtrix3 image processing software package v3.0.2–3.0.3 (https://www.mrtrix.org/) (Tournier et al., 2019). Processing and analysis pipelines for the two openly available datasets are available at https://github.com/fionaEyoung/pipelines.

##### HCP

We accessed 49 scans from the WU‐Minn HCP Young Adult S1200 data release (https://www.humanconnectome.org/study/hcp-young-adult/document/1200-subjects-data-release) (Van Essen et al., 2013). These images have been preprocessed as documented in Glasser et al. (2013). We additionally downsampled them to 2.5 mm isotropic voxels and extracted a subset of 60 directions at $b=1000\;\mathrm{mm}/s^{2}$.

##### TractoInferno

The recently released TractoInferno database (v1.1.1, available at https://openneuro.org/datasets/ds003900/versions/1.1.1) (Poulin et al., 2022), created for the training of machine learning tractography approaches, contains diffusion and T1‐weighted MRI scans for 284 subjects pooled from several studies, accompanied by reference streamline tractography reconstructions. The reference streamline were produced using ensemble tractography and bundles extracted with RecoBundlesX (Garyfallidis et al., 2018) (see Poulin et al., 2022, for full details). Of the 284 subjects included in the full TractoInferno database, we selected the 80 subjects with tractography of all of the AF, CST, IFOF and OR for our study. Nine subjects were excluded from the final analysis due to inadequate non‐linear registration performance resulting in failed in‐house tractography, leaving a final 71 subjects. Diffusion acquisition parameters and preprocessing steps are described in Poulin et al. (2022), and we additionally resampled all data to 2.3 mm isotropic voxels, the lowest resolution present in the dataset and one in line with clinical acquisitions.

##### Clinical

Tract segmentation comparisons are presented for 15 individual scans from eight different subjects from two different institutions. They include four adult glioma subjects acquired in 2009 at the National Hospital for Neurology and Neurosurgery, London (NHNN) (cases 4 and 5 from Mancini et al., 2022; others unpublished data), three paediatric subjects from Great Ormond Street Hospital, London (GOSH) (each with one preoperative and one intraoperative scan), and a mock ‘intraoperative’ scan on a healthy adult volunteer acquired with the GOSH intraoperative DTI protocol and using simulated intraoperative setup (flex‐coils wrapped around the head instead of a head coil, head significantly displaced from scanner isocenter etc.). For acquisition details, see Table 2. All clinical scans involved non‐deforming tumours, in the sense that any lesions did not appreciably displace white matter structures from their typical positions.

**TABLE 2:** Overview of acquisition parameters for the datasets included.

	Clinical				HCP (Glasser et al., 2013; Sotiropoulos et al., 2013)	TractoInferno (Poulin et al., 2022)
	GOSH		NHNN			
	Pre‐op	Intra‐op	Pre‐op	Intra‐op		
*n* subjects	3	4	4	4	49	71
Age	Paediatric	Paediatric (*n* = 3)	Adult		Adult	Adult
		Adult (*n* = 1)				
Indication	Tumour	Tumour (*n* = 3)	Oligodendroglioma (*n* = 2)		Healthy	Healthy
		Healthy (*n* = 1)	Other tumour (*n* = 2)			
*b* values (s/mm^2^)	800 (*n* = 1)	1000	1000	1000	1000	1000 (*n* = 128)
	1000, 2200 (*n* = 2)					700 (*n* = 7)
*n* dirs	15 (*n* = 1)	30	64 (*n* = 3)	30 (*n* = 3)	60^a^	21–128
	60, 60 (*n* = 2)		61 (*n* = 1)	3 × 12 (*n* = 1)		
Voxel size (mm)	1.75 × 1.75 × 2.5 (*n* = 1)	2.5 (*n* = 1)	2.5	2.5 × 2.5 × 2.7	2.5^a^	2.3^a^
	2 × 2 × 2.2 (*n* = 2)	2.3 (*n* = 3)				
Scanner	Philips Ingenia 1.5T (*n* = 1)	Siemens Vida 3T	Siemens Trio 3T	Siemens Espree 1.5T	Siemens 3T ‘Connectome Skyra’	Variable
	Siemens Prisma 3T (*n* = 2)					

^a^ Resampled from original, see text for details.

This study and the use of GOSH clinical data was approved by UCL REC (ID2780/003) and the UCL Institute of Child Health/GOSH joint R&D office (reference 19NI12). Use of NHNN data was approved under retrospective research ethics by the NHNN (University College London Hospitals NHS Foundation Trust) and UCL Institute of Neurology Joint Research Ethics Committee (REC 18/NW/0395, IRAS No: 213920). In addition, the acquisition and use of some NHNN MRI data was also approved by the NHNN (University College London Hospitals NHS Foundation Trust) and UCL Institute of Neurology Joint Research Ethics Committee (REC 12/LO/1977). All clinical data were acquired within the course of routine clinical care, and as no identifying information of any subject is present, there is no need for informed consent. To protect patient confidentiality, clinical data will not be made openly available.

Each dMRI scan was minimally preprocessed with Marchenko‐Pastur principal component analysis denoising (Cordero‐Grande et al., 2019; Veraart et al., 2016) Gibbs‐ringing correction (Kellner et al., 2016) and bias field correction (Smith et al., 2004; Zhang et al., 2001), as implemented in MRtrix3 (Tournier et al., 2019). Preoperative scans additionally had eddy current and motion distortion correction (Andersson & Sotiropoulos, 2016; Smith et al., 2004) (MRtrix3 v3.0.3 and FMRIB Software Library [FSL, https://fsl.fmrib.ox.ac.uk] v6.0) applied, while this step was omitted for intraoperative scans to maintain a clinically realistic timeline. No EPI distortion correction was performed, as it is frequently omitted from clinical pipelines due to lack of requisite reverse phase encoding or field map information and long processing times (Yang et al., 2022).

#### 2.3.2 Compared methods

We considered three alternative segmentation approaches and compared each with the proposed method: Probabilistic streamline tractography, representing the current standard, the deep learning direct segmentation technique TractSeg, and a ‘naive’ atlas registration.

##### Streamline tractography

We ran targeted probabilistic streamline tractography (iFOD2 algorithm, Tournier et al., 2010, from MRtrix3, Tournier et al., 2019, v3.0.3) in each scan using an in‐house ROI strategy (see D.1 for ROI details), with tractography input FODs derived from multi‐shell, multi‐tissue CSD (Jeurissen et al., 2014) with white matter and grey matter tissue compartments. In the clinical dataset, ROIs were placed manually for each subject. For 193 HCP and TractoInferno subjects, manual ROI placement was infeasible. Instead the same ROIs were drawn in MNI152 space aided by the FSL HCP‐1065 DTI template (2023) and transformed to subject space using non‐linear registration (HCP data includes MNI transformation warps, while warps were created for the TractoInferno data using the ANTs registration package v2.4.2 [http://stnava.github.io/ANTs/]; Avants et al., 2011; Tustison & Avants, 2013). This in‐house tractography is subsequently abbreviated to ‘TG’, while the reference TractoInferno bundles are referred to as ‘TGR’. Voxel‐wise segmentations were derived from the streamline reconstructions using track density imaging (Calamante et al., 2010) (more details including thresholds for binary segmentations see Section 2.3.3).

##### TractSeg

TractSeg is a deep learning tract segmentation model which produces volumetric segmentations for 72 tracts directly from fibre orientation distribution peak directions (TractSeg v2.3–2.6, available at https://github.com/MIC-DKFZ/TractSeg). There are two models available: one (‘DKFZ’) trained on modified streamline reconstructions using TractQuerier (Wassermann et al., 2016) as described in, and a second (‘XTRACT’) trained on streamline density maps output by FSL's XTRACT application (Warrington et al., 2020). We compared with both versions, as they feature significant differences in anatomical tract definition. Input peaks were derived from the SSST CSD FODs.

To evaluate tractfinder in a direct comparison with TractSeg using the same training data, we additionally generated new atlases from the reference streamlines available at Wasserthal et al. (2018b) and tested both TractSeg and tractfinder using the corresponding 42 test datasets. The results for this mini experiment can be found in Supplementary Section B.

##### Atlas registration

As well as the full tractfinder method, we compared our results with a ‘naive’ tract atlas approach, taking the density component (first SH coefficient) of the linearly registered tract atlases. This amounts to a segmentation based on prior expectation only, without taking into account the diffusion data.

#### 2.3.3 Comparison metrics

We compared each technique against the others, rather than designating any single technique as ‘ground truth’. Several comparison metrics were computed, to capture different kinds of agreement between segmentations. Dice‐Soerensen similarity coefficient (DSC) (Dice, 1945) is a popular, symmetric measure of segmentation similarity given by

(9) $$\mathrm{DSC}=\frac{2|A\cap B|}{|A|+|B|}$$

for two binary voxel sets A and B. Since DSC is a measure for binary segmentations, it requires the thresholding of continuous‐valued maps such as track density maps and the pseudo‐probability maps produced by tractfinder. Firstly, the conversion from continuous‐valued to binary representation introduces a high degree of ambiguity over the appropriate choice of threshold value. While the simplest approach may be to include all voxels with value 0 in the segmentation, this makes little sense in practice. In the case of tractography, a small number of rogue false positive streamlines can massively increase the extent of the binary segmentation, and in the case of TractSeg, very few voxels actually are assigned a probability of 0. The following thresholds were used throughout, wherever binary segmentations are concerned, are given in Table 3.

**TABLE 3:** Thresholds for binary segmentations.

Method	Threshold	Units
Tractfinder	0.05	[Dimensionless]
Tractography	10	Streamline density
Reference tractography (TractoInferno only)	0	Streamline density
Atlas	0.1	[Dimensionless]
TractSeg	0.5	Probability

Secondly, the binary nature of DSC discounts the additional confidence information present in density and probability maps. The density correlation metric provides an alternative measure of agreement between two continuous valued segmentations with different scales: it is simply the Pearson correlation coefficient between the two sets of voxel values.

**FIGURE 3:**
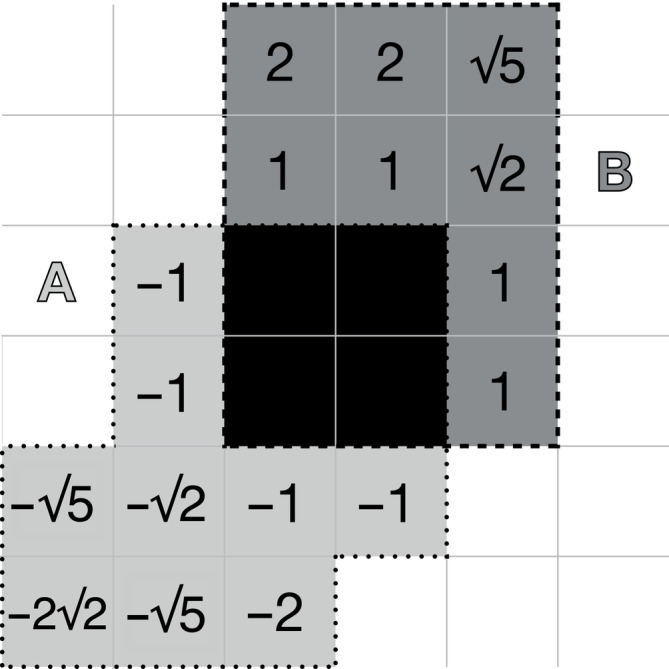
Illustration of regions involved in calculating bundle distance metric. Light grey is A\B, dark grey area is B\A. To compute bundle distance $\mathrm{BD}\left(A,B\right)$ (Equation 10), the mean minimum absolute distance to the intersection (solid black) is taken across all voxels in the two grey areas $\mathrm{BD}\left(A,B\right)=\left(14+4\sqrt{2}+3\sqrt{5}\right)/17=1.55$. To compute the signed bundle distance ${\mathrm{BD}}_{s}\left(A,B\right)$ (Equation 11), distance values in *A* are negated. ${\mathrm{BD}}_{s}\left(A,B\right)=\left(2-2\sqrt{2}-\sqrt{5}\right)/17=-0.18$. The Dice score for these two segmentations would be $\mathrm{DSC}=2\times 4/\left(13+12\right)=0.32$.

In addition to the volumetric overlap and density metrics DSC and density correlation, we measured the volumetric bundle adjacency as defined in Schilling et al. (2021). However, to avoid confusion with the streamline‐based bundle adjacency (Garyfallidis et al., 2012; Radwan et al., 2022; Rheault et al., 2022) metric previously defined in Garyfallidis et al. (2012), and to give more intuitive meaning to the obtained values, we will refer to it as bundle distance BD. It is computed by taking the mean of minimum distances from every non‐overlapping voxel, in each segmentation, to the closest voxel in the other segmentation (Figure 3). Finally, to give a sense of whether the boundary of one segmentation is within or outside that of a second segmentation, we also measured the *signed* bundle distance ${\mathrm{BD}}_{s}$. This metric is asymmetric, with ${\mathrm{BD}}_{s}\left(A,B\right)=-{\mathrm{BD}}_{s}\left(B,A\right)$. Thus BD and ${\mathrm{BD}}_{s}$ are defined as

(10) $$\mathrm{BD}\left(A,B\right)=\frac{\sum_{i\in A\backslash B}d_{i}\left(B\right)+\sum_{i\in B\backslash A}d_{i}\left(A\right)}{|A\Delta B|},$$

(11) $${\mathrm{BD}}_{s}\left(A,B\right)=\frac{\sum_{i\in A\backslash B}-d_{i}\left(B\right)+\sum_{i\in B\backslash A}d_{i}\left(A\right)}{|A\Delta B|},$$

where ∣⋅∣ denotes set cardinality and $d_{i}\left(X\right)$ is the Euclidean distance transform (defined relative to the foreground of segmentation X, i.e. $d_{i}\left(X\right)=0$ when i∈X and $d_{i}\left(X\right)=\mathrm{ij}$ when i∉X and where j∈X is the voxel in X closest to voxel i) of segmentation X at voxel i.

## 3 RESULTS

### 3.1 Atlas

Despite careful manual filtering, it is not possible to completely remove all spurious streamlines from the bundles used to construct the atlases. In particular, SLF and tapetum of the corpus callosum contamination in the optic radiation bundles proved difficult to fully eliminate, despite removing up to 20% of streamlines (Table 1). Nevertheless, sufficient contaminating OFPs were removed to ensure orientations represented in the atlases remained overwhelmingly specific to the target tract (Figure 2).

### 3.2 Processing times

Atlas transformation and inner product computation time per subject for all four tracts and both hemispheres was 24±5s, plus 1–2 min for MSMT‐CSD and 20 s for MNI registration. For TractSeg (DKFZ or XTRACT), mean processing time (for all tracts, 72 for DKFZ and 23 for XTRACT, both hemispheres) was 4:00 ± 1:00 min, plus 15–20 s for SSST‐CSD. For a full processing time breakdown see Table 4.

**TABLE 4:** Measured processing times mean and standard deviation for TractoInferno dataset.

Step	Processing time (s, per subject)	Tractfinder	TractSeg	Atlas	Tractography
Brain masking^a^	3 ± 2	x	x	x	x
Affine MNI registration^a^	20 ± 4	x		x	
Response function^a^	5 ± 3	x	x	x	x
MSMT CSD^a^	110 ± 55	x		x	x
SSST CSD + peaks estimation^a^	18 ± 8		x		
Atlas transformation + inner product (4 tracts, 2 hemispheres)^a^	24 ± 5	x		(x)	
TractSeg DKFZ/XTRACT (72/23 tracts)^a^	240 ± 60		x		
Manual ROI delineation (once for whole dataset)^a^	1200				x
Non‐linear ROI registration + tractography (4 tracts, 2 hemispheres)^b^	334 ± 163				x
Total		2:42 min	4:25 min	<2:42 min	≳27:32 min

*Note*: The tractography pipeline was partially run on a high performance computing cluster, so the reported total time is not representative of a typical setup. Further note that for the present study, tractography ROIs were drawn once for the whole dataset, whereas for clinical datasets manual ROI delineation will have to be repeated for each subject.

^a^ Desktop Mac with 4 GHz Quad‐Core Intel Core i7.

^b^ High performance computing cluster, 1 node per subject, 36 Intel(R) Xeon(R) Gold 6240 CPU @ 2.60 GHz cores per node.

For manual streamline tractography, processing time was not explicitly measured, due to the high variability that comes with manual ROI drawing (between 10 and 25 min for all tracts in a single subject, although this varies significantly between operators). HCP and TractoInferno tractography was run on a high performance computing cluster, taking approximately 10 s per tract (single hemisphere), using 36 CPU cores, and additionally up to 2 min for non‐linear ROI registration (Table 4). However, since the time taken depends greatly on several factors, including number of streamlines to select and streamline acceptance rate (often low in pathological brains due to oedema, deformation etc.), a detailed time analysis for manual tractography is not provided here.

### 3.3 Qualitative results

**FIGURE 4:**
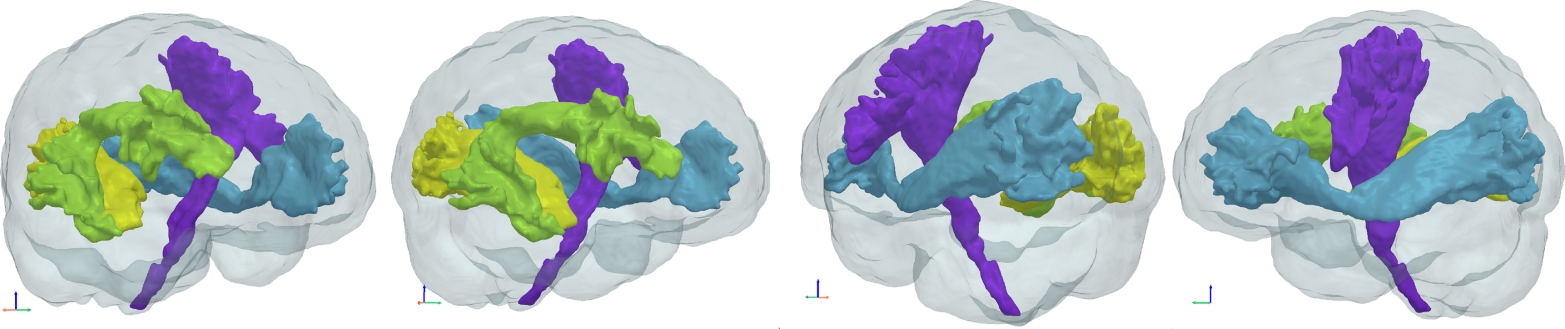
Surface visualisations of tract segmentations obtained using tractfinder in HCP subject 100307. Purple: left corticospinal tract; blue: left inferior fronto‐occipital fasciculus; yellow: right optic radiation; green: right arcuate fasciculus.

**FIGURE 5:**
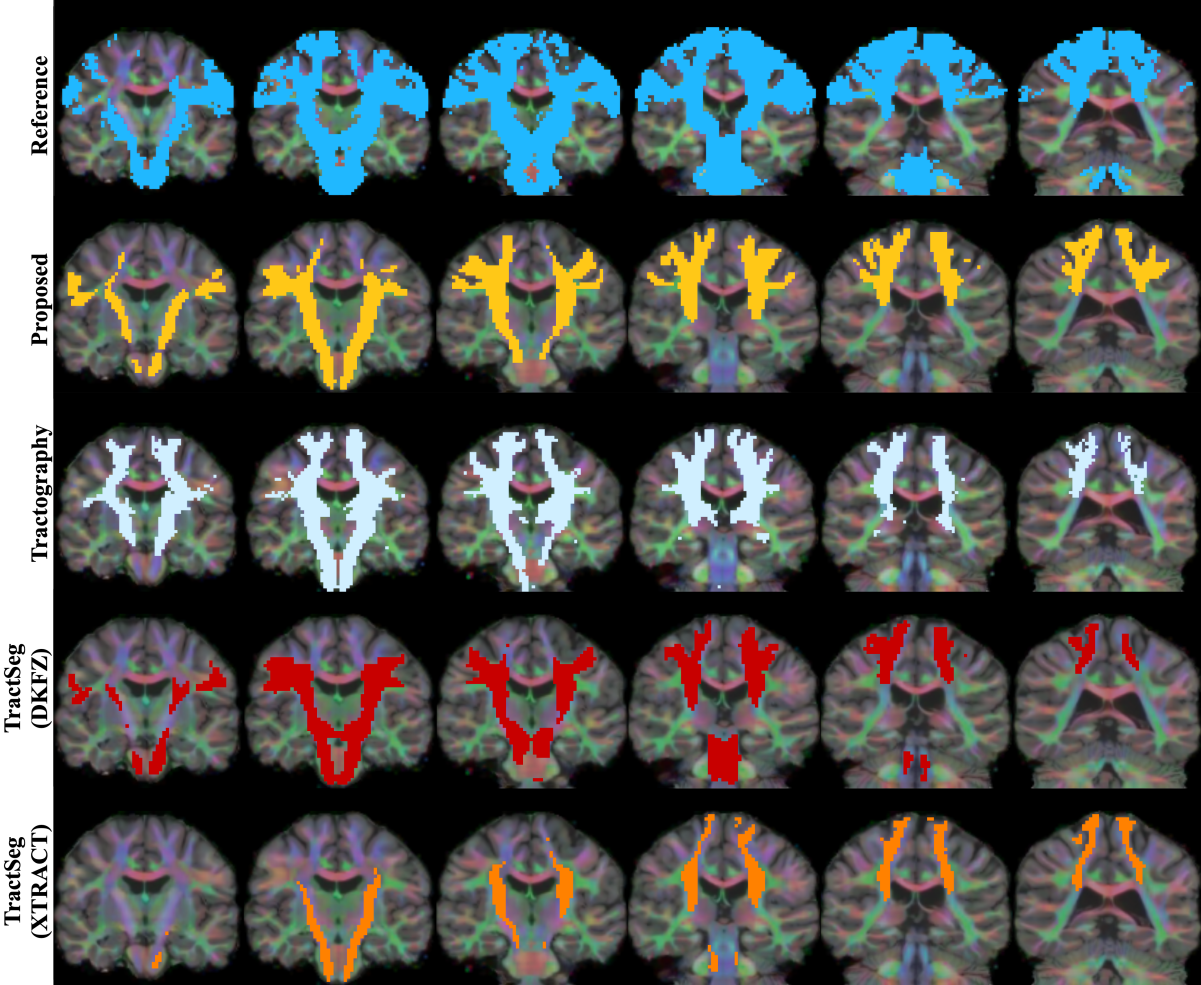
TractoInferno subject 1099: Corticospinal tract. Intensity thresholds are as described in Table 3.

**FIGURE 6:**
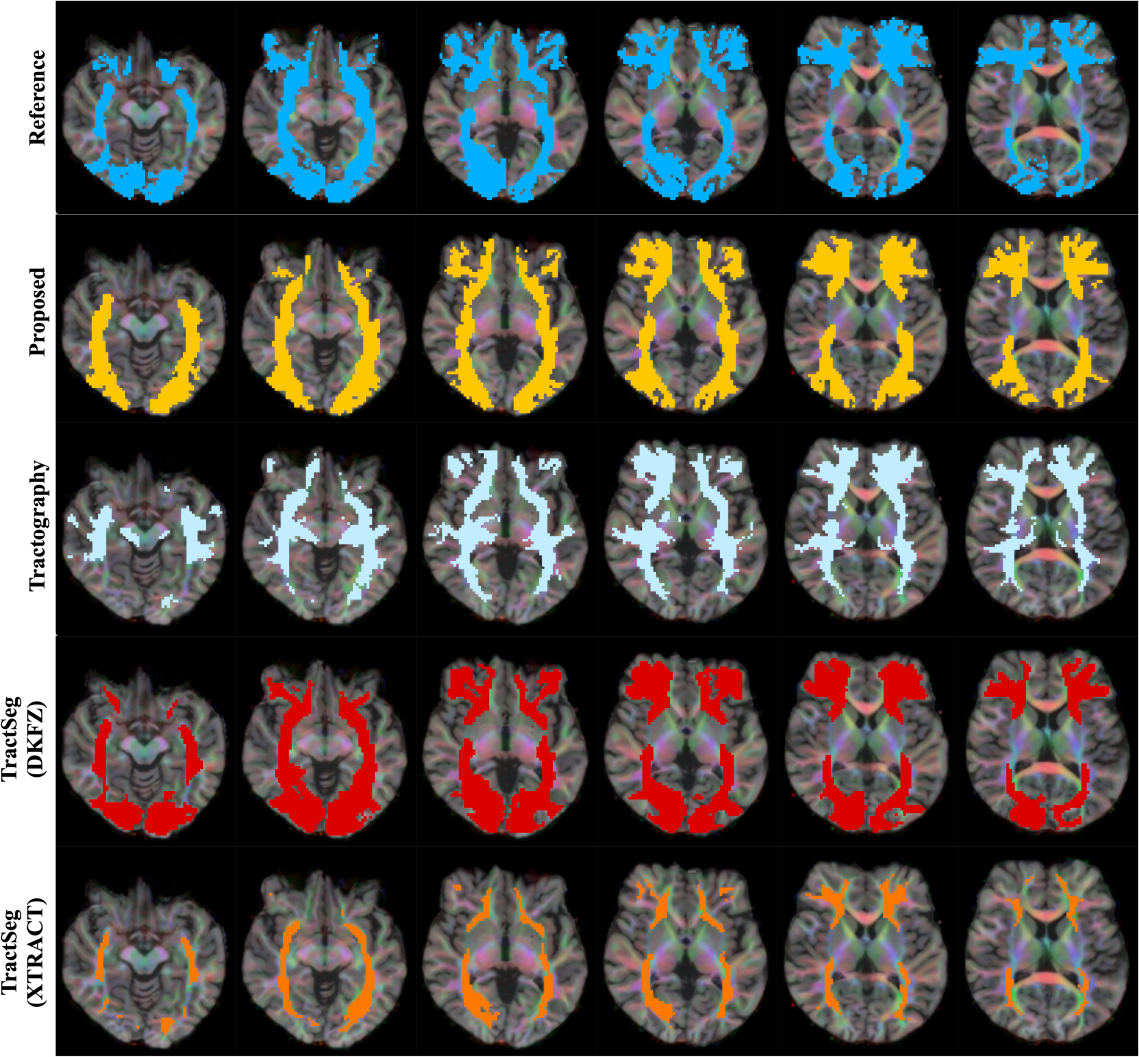
TractoInferno subject 1103: Inferior fronto‐occipital fasciculus. Intensity thresholds are as described in Table 3.

**FIGURE 7:**
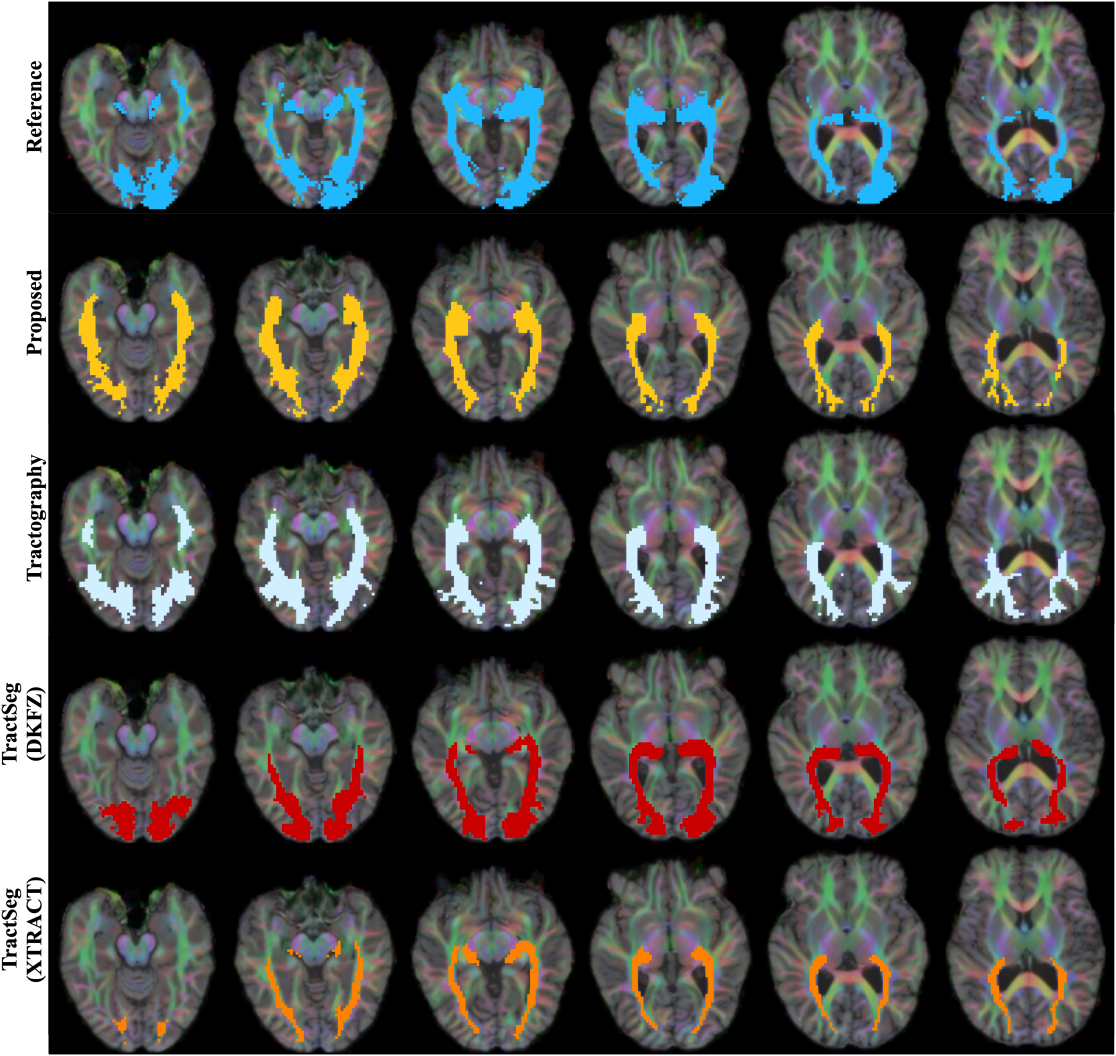
TractoInferno subject 1099: Optic radiations. Intensity thresholds are as described in Table 3.

Qualitative results can be seen in Figures 4, 5, 6, 7 and 11. The raw tract maps typically have values ranging from 0 to 0.5 (in arbitrary units, derived from the magnitudes of FOD and atlas distribution functions). Due to the combined effects of ODF amplitude and orientation information, a low tract map value can have several causes: (a) the FOD amplitude is low, indicating low evidence for white matter tissue in the voxel in question; (b) the atlas amplitude is low, indicating low prior likelihood of the tract being present in that location; (c) the peak orientations between the FOD and atlas are poorly aligned.

Thus combining information from the atlas and data‐derived FODs improves the tract map estimation over the ‘raw’ registered atlas in both the spatial and orientational domain. For example, the TOD atlases have poor definition of gyri and sulci, due to the effect of averaging over many subjects and linear registration. The reduced overall FOD amplitude in grey matter corrects this non‐specificity. And in regions where different white matter structures lie in close proximity, where the atlas can erroneously predict the likely presence of the tract, and FOD amplitude is high, the lack of orientational agreement discounts the presence of the tract of interest in that location.

### 3.4 Quantitative results in healthy data

**FIGURE 8:**
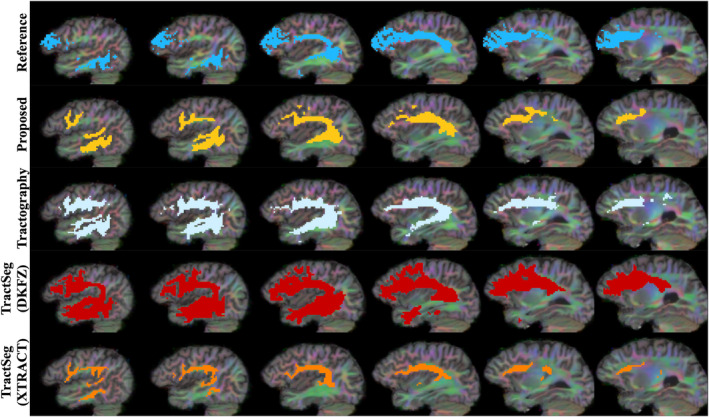
TractoInferno subject 1099: Left arcuate fasciculus. Intensity thresholds are as described in Table 3. See Supplementary Figure E.7 for right hemisphere.

Volumetric and agreement metrics indicate consistent, if not always high, levels of agreement between tractfinder and compared techniques, TractSeg and tractography. Visual assessment reveals that differences in the spatial extent of the segmented tracts accounts for a large part of the discrepancy between methods. This is most apparent in the arcuate fasciculus, where anatomical definitions differ widely (Figure E.7, Figure 8). For example, TractSeg (DKFZ) includes extensive coverage of the frontal and temporal lobe in its AF segmentations, including parts of the primary motor cortex. Conversely in the corticospinal tract, which has a relatively well agreed‐upon domain, segmentation results have much higher volumetric agreement between methods.

The signed bundle distance gives an indication of the nature of disagreement between two techniques where other metrics show little difference. For example, in the HCP dataset and for the arcuate fasciculus, mean bundle distance between the naive atlas and tractography was 5.45 mm and mean bundle distance between TractSeg (DKFZ) and tractography was very similar at 5.41 mm (Supplementary Table 5). However, the signed bundle distances for those same two comparisons were +2.57 mm and −2.68 mm respectively. This indicates that, while if only considering the bundle distance metric, both TractSeg and the atlas appear to agree to a similar degree with tractography, TractSeg actually systematically over‐segments the AF (relative to tractography), while the naive atlas segmentation tends towards under‐segmentation.

Density correlation helps illustrate the cases where the choice of threshold may have a disproportionate influence on subsequent binary comparisons. For example, in the HCP dataset and for the corticospinal tract, mean DSC was 0.69 between tractfinder and tractography and 0.51 between TractSeg (XTRACT) and tractography (a difference of 0.18). For the same two comparisons, the density correlations differed only by 0.04 (0.63 and 0.59) respectively, indicating strong agreement between areas of high confidence (‘density’).

#### 3.4.1 HCP data

**FIGURE 9:**
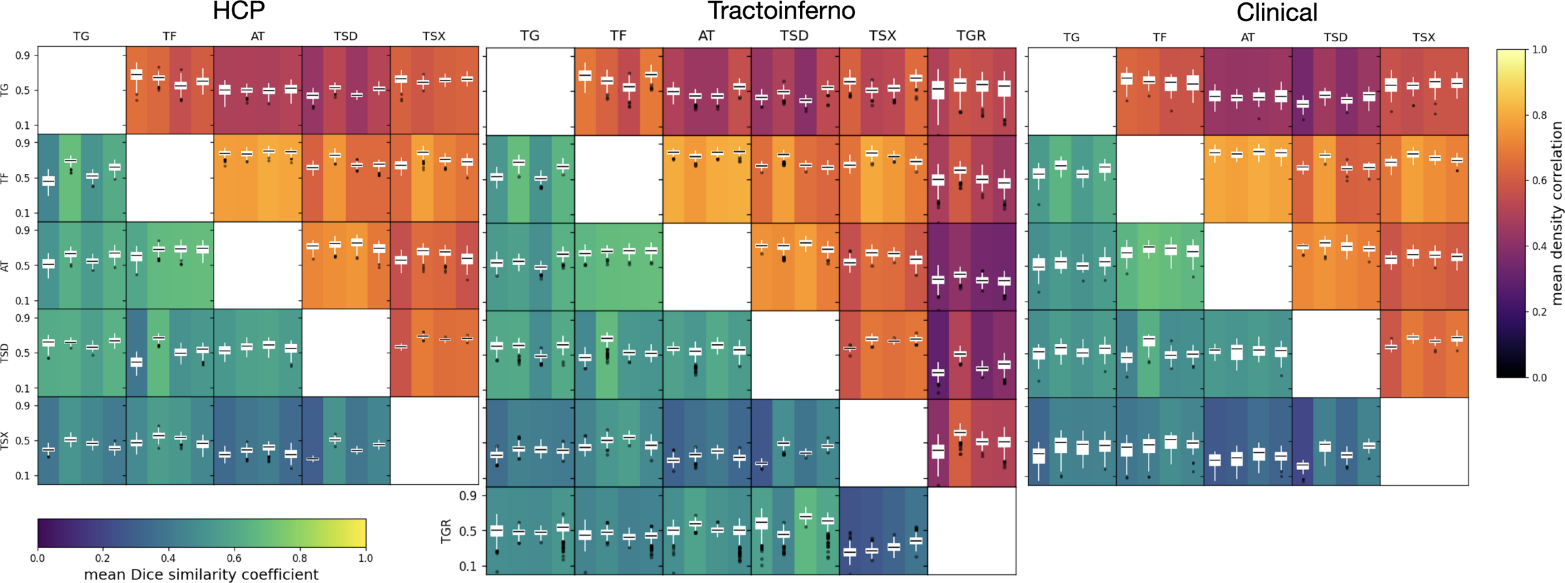
Dice score distributions for each dataset. Each matrix shows the DSCs and density correlations for each method pair in the upper and lower triangles respectively for each tract (from left to right: AF, CST, IFO, OR). Colour represents mean value, with box plots displaying distribution across all subjects. AF, arcuate fasciculus; AT, atlas; CST, corticospinal tract; IFO, inferior fronto‐occipital fasciculus; OR, optic radiation; TF, tractfinder (proposed); TG, in‐house tractography; TGR, reference tractography (TractoInferno streamlines); TSD, TractSeg (DKFZ); TSX, TractSeg (XTRACT).

Volume surface visualisations obtained with tractfinder are shown for the four tracts in a single HCP subject in Figure 4. Figure 9 gives an indication of how the five different segmentation methods compare, across all HCP dataset subjects. There is considerable variance between tracts, however some observations remain consistent.

DSCs are low across the board for the arcuate fasciculus, owing to the dramatically different spatial extents of the segmentations. For the corticospinal tracts, tractfinder agrees well with each of the other methods, measured by both DSC and density correlation. Agreement is similarly high in the optic radiations, with slightly lower DSCs compared to the two TractSeg methods, which tend to include more thalamus and a lesser extent of Meyer's loop.

Tractfinder segmentations are highly consistent, with comparison metrics with alternative methods varying by little across subjects (Supplementary Table 5).

#### 3.4.2 TractoInferno

Qualitative results for a representative subject (identified as the only subject within the top 30 smallest deviations from the mean scores for all three of bundle distance, DSC and density correlation) are shown in Supplementary Figures E.7–E.10.

**FIGURE 10:**
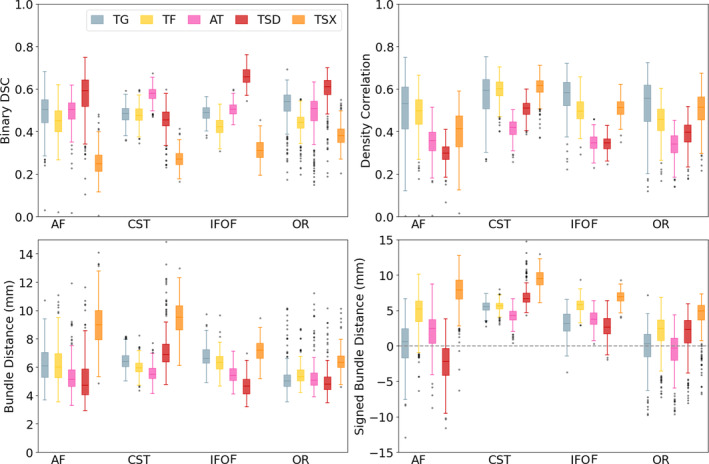
Comparison of each other segmentation (including in‐house tractography) method against reference tractography in the TractoInferno dataset. Bundle distance is equivalent to *bundle adjacency* in Schilling et al. (2021). AF, arcuate fasciculus; AT, atlas; CST, corticospinal tract; IFO, inferior fronto‐occipital fasciculus; OR, optic radiation; TF, tractfinder (proposed); TGR, reference tractography (TractoInferno streamlines); TG, in‐house tractography; TSD, TractSeg (DKFZ); TSX, TractSeg (XTRACT).

Figure 10 compares each studied method against the reference streamline bundles in the TractoInferno dataset. Noticeably, the differences in scores within a single method, between different tracts, are in places greater than the differences between methods within a tract. For example, the DSC scores for the CST are similar for tractfinder and TractSeg (DKFZ) (0.48 and 0.45 on average respectively), however the DSCs of TractSeg (DKFZ) are markedly different between the CST and OR (0.45 and 0.59 on average respectively). These differences highlight the difficulty in assessing the ‘accuracy’ of white matter segmentation methods given limited consensus on the precise anatomical definitions of different pathways. Density correlation values for tractfinder were on par with TractSeg (XTRACT) in all tracts, with the exception of the OR, while density correlation was higher than TractSeg (DKFZ) in all tracts. DSC scores were highest for TractSeg (DKFZ) in the IFO and AF, and equal between tractography, tractfinder and TractSeg (DKFZ) for the CST. The Dice score is less sensitive to errors in the segmentation of large volumes (Eelbode et al., 2020; Taha & Hanbury, 2015), which explains why TractSeg scores well on tracts like the AF and IFO, which cover large volumes and can, in some definitions, extend to many different cortical regions. The results in Figure 10 are consistent with the comparisons between TractSeg and RecoBundles published. There, a mean DSC of between 0.58 and 0.67 across all tracts was reported. Our measured DSCs between TractSeg (DKFZ) and reference tractography (which is based on RecoBundlesX (Garyfallidis et al., 2018)) range between 0.45 and 0.59 across the four tracts studied (Supplementary Table 7).

From Figure 10, it is apparent that comparisons with TractoInferno reference streamlines yield a large number of outliers and low scores. Further investigation into these outliers revealed numerous subjects with incomplete or highly asymmetric bundles. For example, in several cases, optic radiation streamlines only reach the superior portion of the occipital lobe (Supplementary Figure E.8a). In others, the right arcuate fasciculus is significantly smaller than the left (Supplementary Figure E.8b). There is lower variability in the pairwise comparisons between the other four methods: the results for tractfinder and TractSeg remain in more consistent agreement with each other across the TractoInferno dataset (Figure 9).

### 3.5 Results in clinical data

For the present analysis we included clinical scans with non‐deforming lesions, meaning the orientation atlas could be registered to the target image using only affine registration without the need for tumour deformation modelling. For qualitative results in clinical scans featuring deforming lesions, see Young et al. (2022).

In the clinical dataset, mean values of agreement with other segmentation methods were consistent with those in the ideal, healthy datasets, while variability between subjects was higher (Figure 9). Again, comparisons between the segmentation methods vary significantly between tracts. The size of this dataset is far smaller than the other two, but even so the results are consistent with those seen for the larger, healthy subject datasets.

**FIGURE 11:**
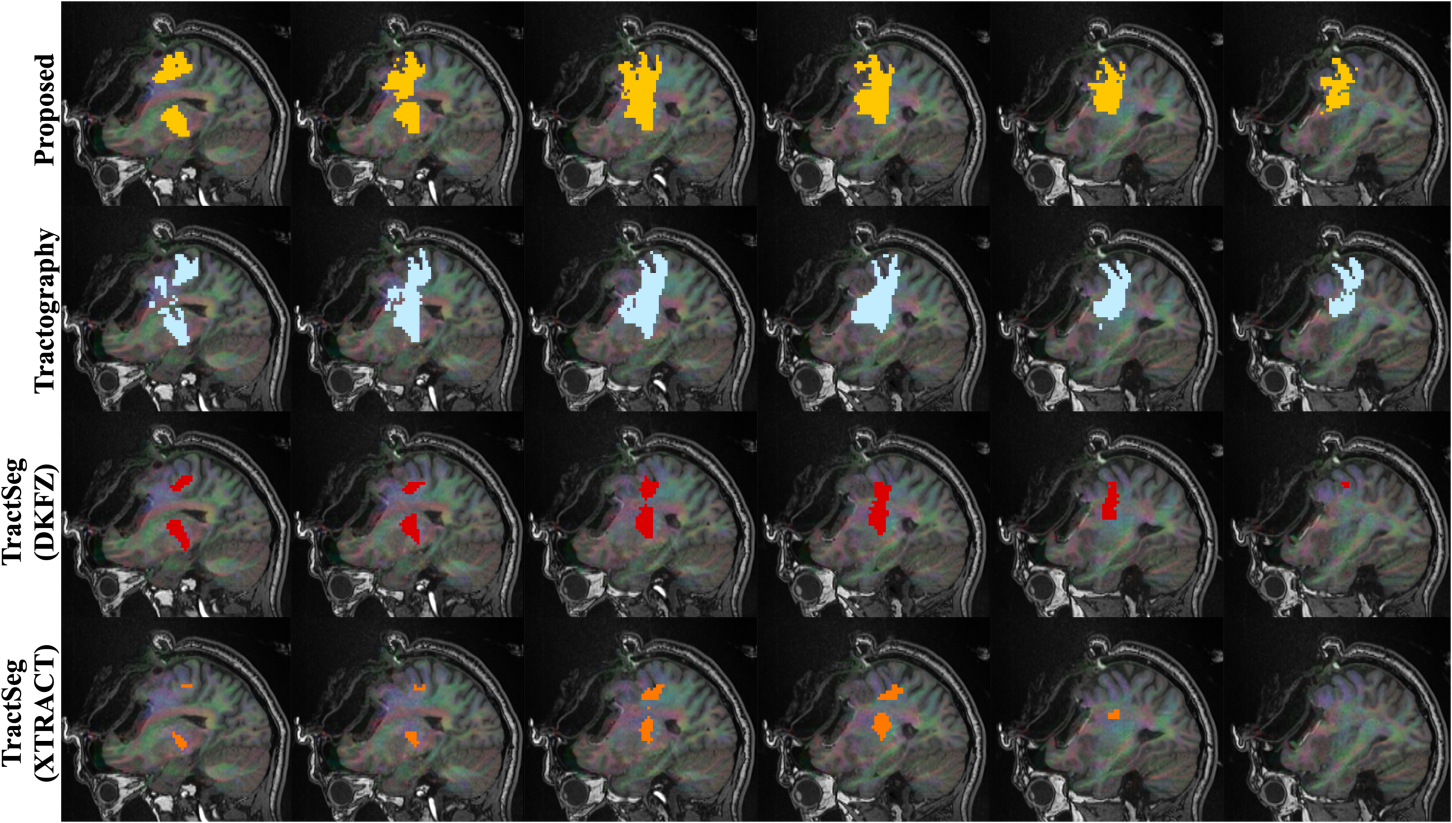
Right corticospinal tract segmentation maps for all methods in NHNN subject 5, intraoperative scan. Intensity thresholds are as described in Table 3.

**FIGURE 12:**
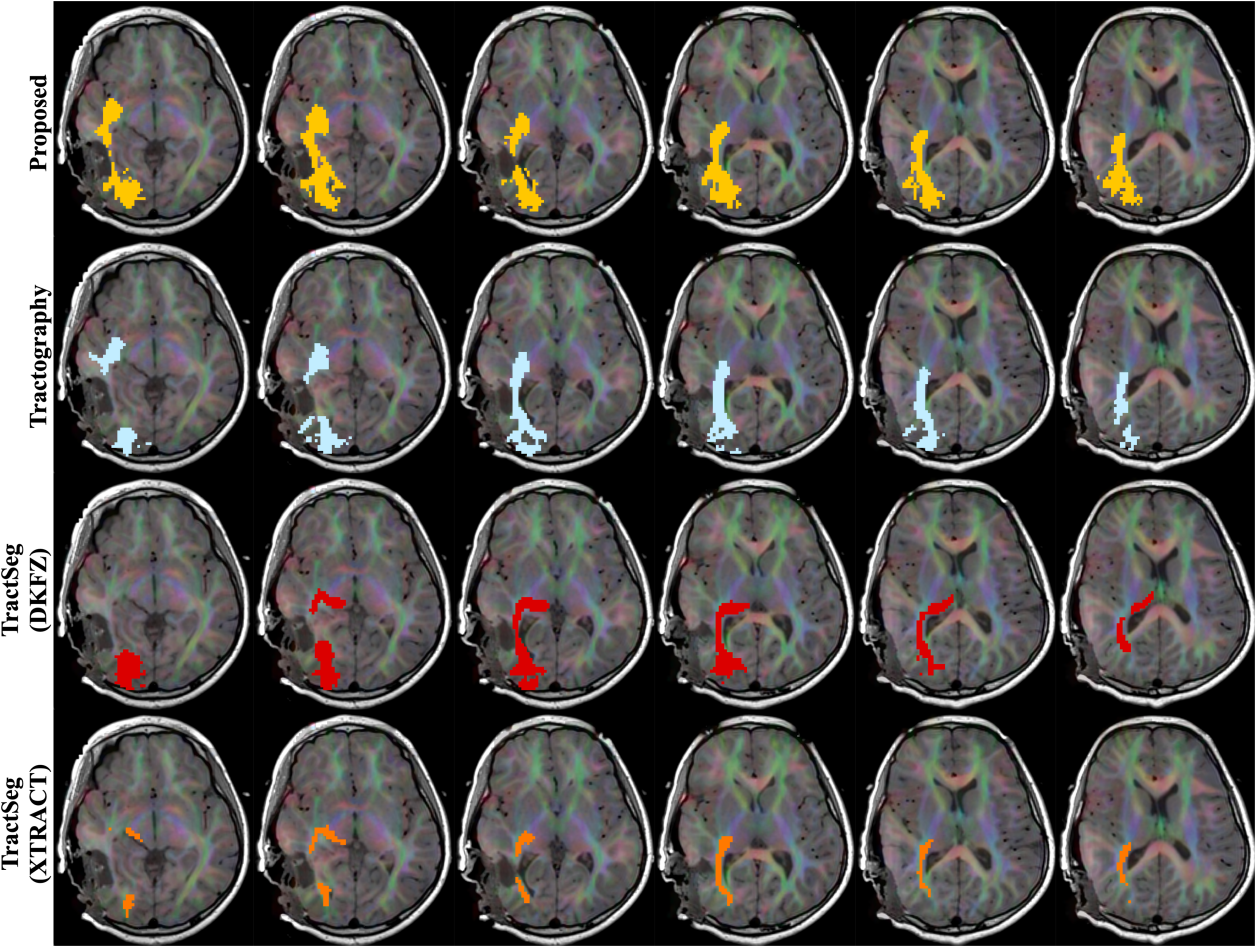
Right optic radiation segmentation maps for all methods in GOSH subject 3, intraoperative scan. Intensity thresholds are as described in Table 3.

Two example clinical subjects, one adult and one paediatric, are displayed in Figure 11 and Figure 12. In Figure 11, a sagittal view displays the interaction between the surgical resection cavity and the CST. Here our proposed method maps the CST in relatively close proximity to the resection site, where the TractSeg segmentations are far more conservative, potentially missing CST locations influenced by oedema or other tumour effects. In Figure 12, the extent of Meyer's loop depicted by tractography is similarly included in the proposed segmentation, but absent from the TractSeg results.

When the results for the clinical dataset were split on hospital/age group (paediatric or adult), no appreciable difference in results was observed (data not shown). Equally, no systematic difference was observed between intraoperative and preoperative datasets. The mean score results for all tracts and comparisons are given in Supplementary Table 6.

## 4 DISCUSSION

We have presented here a full methodological description and validation of a novel white matter segmentation technique, which can produce consistent results and be run fully automatically (although the user can manually adjust threshold values if required). Accuracy, so far as can be measured, is comparable with alternative techniques, with differences in comparison metrics being driven primarily by disagreements in tract definitions, rather than by methodological performance.

The inner product between the orientation atlas and target FOD image provides an intuitive map of tract location and is computationally straightforward. An advantage of tractfinder over a deep learning method is the element of explainability that is provided by the orientation atlas and subsequent combination with the data. The simple mathematical formulation affords an intuitive understanding of why a given voxel is included in a segmentation, and can be visualised along with the subjects FODs for additional clarity.

A key finding in Toescu et al. (2020) was the logistical barrier to deployment of tractography in neurosurgical units in the United Kingdom, as well as a lack of knowledge among neurosurgeons of the underlying choices in fibre modelling and tracking algorithms involved, which could lead to flawed interpretations of tractography reconstructions. We hope that the use of a predefined tract atlas in combination with readily automated registration and dMRI processing tools will go some way towards reducing that logistical burden, as well as aiding interpretation of the results.

It is important to note that for white matter segmentation, there is currently no single optimal technique: each has individual strengths and weaknesses and are appropriate for different contexts, depending on constraints on processing time, computer power, operator expertise as well as the context of application (e.g. whether streamlines are required or voxel‐wise segmentations). Tractfinder, as well as being generally applicable to healthy datasets, has been developed specifically with a neurosurgical context in mind, and can flexibly accommodate minor tumour distortions and epilepsy pathologies out‐of‐the‐box, and larger distortions with additional adjustment as described in Young et al. (2022). Notably, the ability to generalise to subjects with space‐occupying lesions is not dependent on additional expertly annotated tumour training datasets for atlas creation, as the tumour deformation modelling can be done at the point of application in a subject specific manner.

A further advantage of the proposed atlases is that they require relatively few training scans (Young et al., 2023). This allows for easy generalisation to other tracts and, where preferred, reconstruction of existing atlases with different anatomical definitions. By contrast, re‐training a deep learning model to recognise new or differently defined tracts requires hundreds of training subjects and corresponding manual tract annotations. If a research or surgical team already espouses particular tractography protocols for their commonly reconstructed tracts, then an atlas using existing sample reconstructions can easily be built and used to automate the tract segmentation process, without needing to adopt different tract definitions.

Taken together, the flexibility of the proposed framework makes it ideal for use in clinical, and in particular intraoperative, as well as low‐resource research environments. Alternative atlases can be constructed with little computational burden to suit local needs. Some space‐occupying lesions may be accounted for without the need for any additional annotated clinical training data. And finally, time savings of even a few minutes can make all the difference in a surgical setting, where clinical decisions may be made on the fly as intraoperative scans are acquired.

### 4.1 Comparison with alternative segmentation methods

Comparing methods as varied in approach as direct atlas‐based segmentation, deep learning inference and streamline tractography makes objective comparison difficult. Qualitatively, the spatial coverage for all four tracts shown is well‐matched with streamlines, while TractSeg (DKFZ) generally produces broader segmentations. There is also qualitative evidence that tractfinder can produce reconstructions with sensitivity more comparable to that of tractography than TractSeg in situations of surgical relevance, such as the proximity of the CST to the resection site in Figure 11, or the extent of Meyer's loop, a structure with frequent significance for postoperative visual field deficits (Daga et al., 2012; Lacerda et al., 2020; Winston et al., 2014), in Figure 12.

Multiple quantitative volumetric similarity metrics are presented here to give a rounded picture of the differences in results, as no single measure can helpfully capture all aspects of complex tract segmentations (Rheault et al., 2020).

We compared tractfinder and three other segmentation approaches to the reference streamline bundles in the TractoInferno dataset. Among all metrics and tracts, the large range of values indicates either a high degree of variability in the reference streamline bundles, or a low level of robustness in all of the investigated methods. Some inconsistencies in the reference bundles (Supplementary Figure E.8) resulted in a large number of outliers in comparisons with all four other methods. These results have been included here as we wanted to take this dataset of reference bundles, which has been published for the purpose of training and validating machine learning tractography algorithms, ‘at face value’. They demonstrate how even with diligent manual quality control, achieving consistent, reproducible white matter segmentation results in hundreds of subjects using streamline tractography remains extremely difficult.

In Figure 10, we compared our approach and three other segmentations with the TractoInferno reference bundles. However, we should avoid interpreting these comparisons as measures of ‘accuracy’ for the respective methods. Take, for example, the higher binary DSC (0.58) obtained by a naive atlas registration, without incorporating any subject diffusion information, compared to TractSeg (DKFZ) (0.45) for the CST. Should we therefore conclude that a simple affine registration of an atlas, without any incorporation of subject specific diffusion information, is ‘better’ in this case than a deep learning inference based on fibre orientation peak directions? Consider also the large differences between the TractSeg (DKFZ) and TractSeg (XTRACT) results, which demonstrate how the underlying tract definitions from which a prior is derived (e.g. an atlas, deep learning model, etc.) is the driving factor in dice score ‘accuracy’. Numerical metrics can thus highlight tendencies and help determine which methods perform consistently, while visual assessment remains the only way of gauging anatomical accuracy.

We also compared segmentation approaches in two other datasets with no reference bundles: a set of HCP subjects and a selection of clinical subjects. The problem of defining reference bundles for the validation of tract segmentation still needs to be properly addressed by the diffusion imaging community. It is a two‐fold problem: a lack of both a sufficiently accurate and reliable method and of agreed upon anatomical definitions (see below). Some tract segmentation studies, for example, the deep learning methods Neuro4Neuro (Li et al., 2020) and TractSeg, define their own reference bundles used for both model training and evaluation. While the in‐house tractography results presented for the clinical and HCP datasets represent the current standard process, treating them as reference bundles could have produced biased interpretations, as the same ROI strategy and anatomical interpretation were employed to produce the tract atlases. Instead we present only pairwise comparisons between all techniques and qualitative results. It is for the same reasoning that we opted not to include the publicly available bundles for the 105 HCP subjects on which TractSeg (DKFZ) was trained as additional reference bundles for the HCP dataset in our analysis.

In the HCP dataset, tractfinder agrees strongly with the other methods, particularly in the corticospinal tracts. TractSeg (XTRACT) stands out as having low binary DSC scores when compared with the other methods. Comparisons with streamline tractography and non‐tractography methods generally exhibit very low gDSC values. This is presumed to be due to the extreme density bias common in TDI maps (Rheault, Poulin, et al., 2020), with values within a relatively small central portion of the tract being orders of magnitude greater than in the periphery.

### 4.2 Data requirements

We have previously investigated the effect of reduced number of diffusion weighting directions and different fibre orientation distribution reconstruction approaches on segmentation stability for the segmentation methods analysed here (Young & Clayden, 2022). Segmentation results using tractfinder remained consistent within a subject even with decreasing data acquisition quality, from 120 directions and two (nonzero) *b*‐value shells down to as few as 12 directions at b=1000. The ideal acquisition is at least 60 diffusion directions at b=1000, although fully acceptable results can be obtained with just 30 directions, as in the presented intraoperative data. The full breakdown of data quality and FOD modelling effects can be found in Young and Clayden (2022).

### 4.3 Limitations

An atlas‐based tract segmentation method such as the one proposed here is not suitable for ‘exploratory’ connectivity studies, where tract segmentations and connections are sought without imposing any prior expectations. Instead, this approach is more suited for segmenting known pathways, and for when rapid and robust segmentation is a priority.

Owing to the intensive process of constructing a new atlas, we opted to focus on validating our approach on a few tracts before moving on to constructing further atlases. The availability of atlases for the CST, AF, IFOF, and OR only limits the applicability of our technique to those four tracts for the time being, whereas many alternative methods can segment tens of different pathways at a time. The construction of atlases for other commonly studied pathways, such as the UF, will be the subject of future development. However, users can easily construct additional custom atlases using only 10–20 training samples, and code to do so is available online: https://github.com/fionaEyoung/tractfinder.

The current approach of taking the inner product of the two angular distributions produces good results, is computationally straightforward, and has intuitive meaning. However, a potential drawback, depending on the desired information to be provided by tract mapping, is the behaviour in the presence of multiple fibre populations. The presence of crossing fibres will reduce the map amplitude, even if one of the FOD fibre populations aligns well with the atlas, as the presence of the crossing fibre population reduces the overall inner product value. If the objective is to obtain a likelihood score for a particular tract, regardless of whether or not the tract is sharing a voxel with another fibre population, then the current inner product framework, which slightly penalises crossing fibre voxels over single fibre ones, is inadequate. However, the signal loss due to crossing fibres is not severe and remains well above threshold for inclusion in the segmentation, and binary segmentation is therefore unaffected by this feature of the inner product approach.

Tractfinder requires an additional registration step and relies on good alignment of the atlas to subject data, although in this regard affine registration is sufficient. While registration does not significantly add to the processing time of the pipeline overall, it nevertheless introduces an additional source of error and variability. In healthy data, registration tools are largely robust, however in some subjects, including those featuring pathology or who were scanned with a non‐standard head orientation (as in many intraoperative cases), registration can prove less robust, required manual intervention and parameter tweaking.

Finally, tractfinder relies on HARDI diffusion data, and thus does not benefit from the shorter scan‐times afforded by the simpler diffusion tensor acquisitions which are sufficient for the diffusion tensor‐based tractography tools available on commercial neuronavigation tools.

## 5 CONCLUSION

Diffusion MRI holds great potential for neurosurgical navigation, a potential which remains accessible only to select institutions with the requisite means, staff and equipment to fully exploit it via advanced neuroimaging techniques including multi‐fibre orientation modelling and probabilistic tractography. Obtaining useful streamline tractography results from suboptimal datasets is especially difficult and time‐consuming. We have developed a novel tract segmentation technique for rapidly mapping important fibre tracts with the aid of tract orientation atlases. Results are highly consistent with targeted probabilistic tractography and comparable with the deep learning technique TractSeg, although considerable differences in anatomical definitions confound interpretations of object ‘accuracy’. The simplicity and efficiency of the proposed methods could bring advanced diffusion MRI processing closer to widespread clinical adoption, without compromising on segmentation quality in comparison to streamline tractography.

## FUNDING INFORMATION

This work is supported by the EPSRC‐funded UCL Centre for Doctoral Training in Intelligent, Integrated Imaging in Healthcare (i4health) (EP/S021930/1). In addition, all research at Great Ormond Street Hospital NHS Foundation Trust and UCL Great Ormond Street Institute of Child Health is made possible by the Department of Health's NIHR‐funded Great Ormond Street Hospital Biomedical Research Centre. Some of the NHNN data acquisition was funded by a grant provided by the NIHR UCL/UCLH Comprehensive Biomedical Research Centre (BRC) (Project Award reference 74). LM was supported, at the time of NHNN data acquisition, by the NIHR UCL/UCLH BRC. The views expressed are those of the authors and not necessarily those of the NHS, the NIHR or the Department of Health.

## CONFLICT OF INTEREST STATEMENT

The authors declare the following financial interests/personal relationships which may be considered as potential competing interests: Fiona Young, Jonathan D. Clayden, Kristian Aquilina and Chris A. Clark have patent pending to UCL Business PLC. All other authors declare no known competing financial interests or personal relationships that could have appeared to influence the work reported in this paper.

## Supporting information


**Data S1.** Supporting Information.

## Data Availability

Scripts for running tractfinder and generating custom tract atlases are available at https://github.com/fionaEyoung/tractfinder. Tract orientation atlases (and corresponding training streamlines) for the AF, CST, IFOF and OR are openly available for non‐commercial use: https://doi.org/10.5281/zenodo.10149873. This study utilised data from the Human Connectome Project (HCP) and TractoInferno database, publicly available at https://www.humanconnectome.org/study/hcp-young-adult/document/1200-subjects-data-release and https://openneuro.org/datasets/ds003900 respectively. Clinical data from Great Ormond Street Hospital and the National Hospital for Neurology and Neurosurgery cannot be publicly released to maintain patient confidentiality. Processing pipelines and code scripts used to compare segmentations and produce summary figures are available at https://github.com/fionaEyoung/pipelines.
