# Supplementary material for: Fibre orientation atlas guided rapid segmentation of white matter tracts
Source: Hum Brain Mapp. 2024 Jan 30;45(2):e26578. doi: 10.1002/hbm.26578 (PMC10826637; doi:10.1002/hbm.26578)
Supplement: Supplementary file 1 — Data S1. Supporting Information. [file HBM-45-e26578-s001.pdf]

Supplementary material

A Atlas registration: affine versus non-linear

Affine registration is faster, more robust, and has fewer parameters that may need manual adjustment that non-linear registration. There is, in principle, nothing to stop the user choosing to use non-linear registration to transform the atlas into target subject space. We compared the results when using either affine (FMRIB's flirt tool) or non-linear (ANTs registration package Symmetric Normalisation algorithm) registration in the Tractoinferno dataset. (Note that the atlases are unchanged from those used in the main text, meaning the generation of the atlases still involves only affine registration between training subjects.) Figure A.1 demonstrates that there is no advantage to using nonlinear registration in healthy data, as all results were unchanged across all tracts and performance metrics.

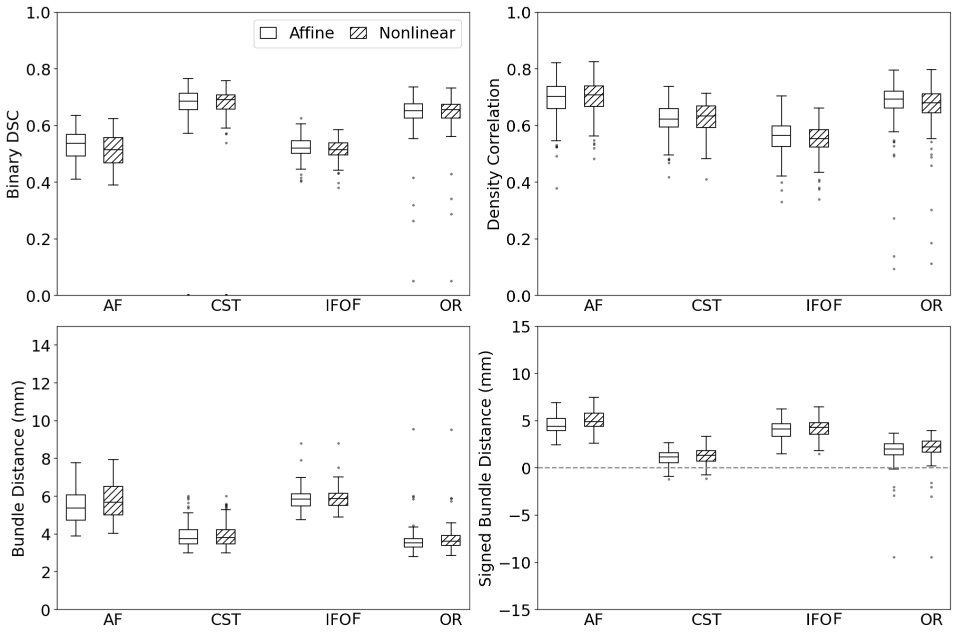

Figure A.1 Difference in tractfinder performance when using either affine (plain) or non-linear (hatched) atlas registration, compared with targeted ROI tractography (see Supplementary section D.1), in the Tractoinferno dataset. For binary measures, a threshold of 0.05 was applied.

B Direct comparison with TractSeg

A core feature of tractfinder is the creation of tract atlases from carefully filtered streamline bundles based on strict anatomical definitions. Nevertheless, the use of custom training data makes the comparison between benchmarks methods difficult, as differences in anatomical definitions dominate the results. In order to directly compare tractfinder with another method, we created additional atlases from 16 randomly selected subjects of the TractSeg training data (used to train the default DKFZ weights, TractSeg v2.3-2.6, available at https://github.com/MIC-DKFZ/TractSeg) and reference streamlines, and tested the atlases on the same 42 test subjects used in the original TractSeg evaluation (subject IDs available at Wasserthal, Neher, and Maier-Hein (2018a))

The results are displayed in Figure B.2. When considering both the Dice score and density correlation metrics, neither method stands out as being more accurate when trained and tested against the same data.

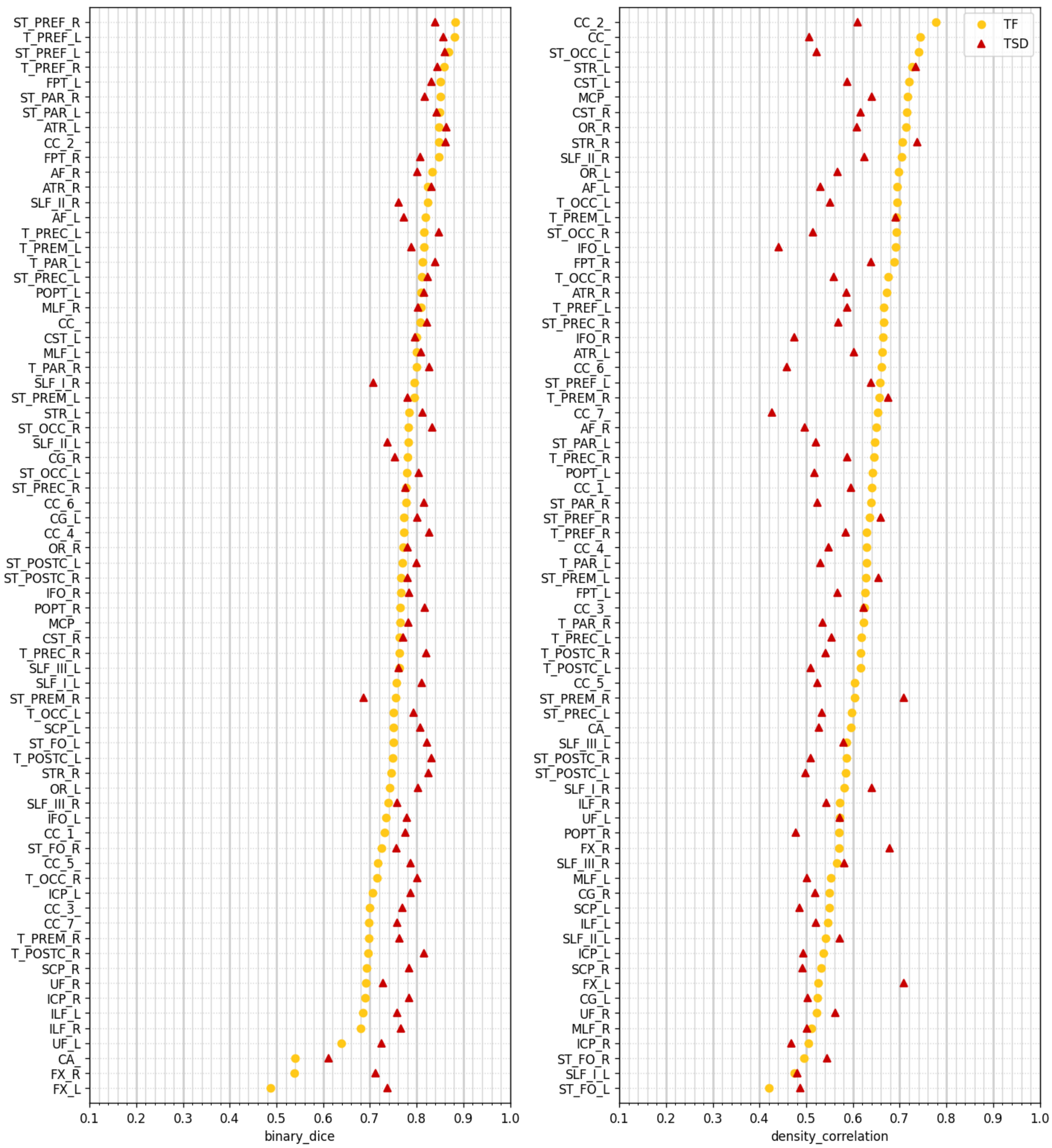

Figure B.2 Dice similarity coefficients (left) and density correlations (right) of tractfinder (yellow) and TractSeg (red) trained and test on the DKFZ reference tracts.(Wasserthal, Neher, and Maier-Hein, 2018b) This figure can be directly compared with Figure 6. in Wasserthal, Neher, and Maier-Hein (2018b), see Wasserthal, Neher, and Maier-Hein (2018a) for full tract names.

C Notes on tract definitions

Corticospinal tract

Standardised white matter atlases and tractography protocols varyingly describe the corticospinal and pyramidal tracts. These two terms are often used interchangeably in tractography-oriented publications, while in anatomical terms they are distinct: The corticospinal (CST) and pyramidal tracts (PyT) are both descending motor pathways, with the PyT encompassing both the CST and the corticobulbar tract, which controls movement of the head, neck and face via the cranial nerves. Tractography studies and related white matter segmentation research frequently conflate the major descending (motor) and ascending (sensory pathways). This is evident in two main regions. Firstly, the inclusion of the medial lemniscus is frequently seen in PyT or CST segmentations (usually as it is not explicitly excluded, rather than being actively included). This includes TractSeg (and associated reference streamline bundles), XTRACT to some extent, and TractoInferno. By contrast, the tractography protocol employed in this research includes an exclusion mask on the medial lemniscus.

Secondly, while it has been suggested that the primary motor cortex can reside in the post-central gyrus,(Kumar et al., 2009) it is generally accepted that the somatosensory cortex is located in the latter, while the motor areas are in the precentral gyri. However, particularly with probabilistic tractography, it is near impossible to constrain streamlines exiting the internal capsule into the fanning corona radiata to one side of the central sulcus, without additional exclusion planes or the use of cortical target regions, which are especially time-consuming to produce, whether manually or through automatic parcellation. Thus streamline-based CST segmentations often contain parts of the somatosensory cortex while others, such as those utilising cortical parcellation-derived target regions, will be restricted to the motor cortex.

The tractfinder CST atlas streamlines were obtained using Freesurfer parcellations (Desikan et al., 2006; Fischl et al., 2002) of the primary motor cortex, as are the TractSeg reference bundles. TractInferno reference bundles for the pyramidal tracts include sensory cortex.

Optic radiation

When it comes to the course of the optic radiations through the sagittal stratum and posterior termination in the occipital lobes, there is no disagreement between segmentation approaches. However, there remain significant differences in the regions of the lateral geniculate nucleus (LGN) and Meyer's loop. The LGN is a small nucleus of the thalamus from which the neurons of the OR originate. Its localisation on MRI images is not straightforward, and due to the complex arrangement of white matter structures in the upper midbrain and thalamus regions, it is easy for streamlines to extend into the entire posterior thalamus and fornix and even descend into the brainstem. This contributes to often broad OR segmentations in the thalamic portion at the start of the tract. Secondly, the full anterior extent of Meyer's loop is often not reconstructed by tractography, due to the extreme and tight curvature.(Lilja and Nilsson, 2015; Chamberland, Tax, and Jones, 2018)

Arcuate fasciculus and inferior fronto-occipital fasciculus

Of the tracts studied in this work, the arcuate fasciculus (AF) and inferior fronto-occipital fasciculus (IFOF) exhibit the most extreme variability in segmented anatomical extent. This is partially owing to disagreements in definition, as they are both association pathways, making agreements about their function and precise cortical targets hard to find. For example, while the general consensus is that the AF connects the temporal

and frontal language areas, XTRACT follows the "three part" paradigm(Catani, Jones, and Ffytche, 2005) which includes a third cortical termination region in the supramarginal gyrus, or inferior parietal cortex. There are also controversies about whether the IFOF terminates in the parietal and temporal, in addition to the occipital lobes,(Martino et al., 2010; Forkel et al., 2014; Weiller et al., 2021) with some suggesting it be subdivided into two components based on these posterior terminations.(Martino et al., 2010; Sarubbo et al., 2013; Rollans and Cummine, 2018) Furthermore, unless cortical parcellation derived termination masks are utilised, it is practically impossible to constrain streamlines to a compact pathway, with bundles frequently terminating within large swathes of the frontal and temporal lobes.

D Tractography ROIs and parameters

D.1 ROI definitions

The following ROI strategies were used for atlas constructions and subsequent validation tractography (differences between the two specified where applicable). Visualisations of each ROI are shown on MNI152 template in Figures D.3, D.4 and D.6.

Arcuate fasciculus

Seed White matter medial of angular gyrus, visible on coronal views of colour fractional anisotropy maps as a "green triangle", drawn on the coronal plane. Level of coronal plane selected from sagittal view by locating the central sulcus (Fig. D.3, arrow).

Include Descending section of the arcuate fasciculus, drawn on the axial plane

Exclude Exclusion ROIs targeting: midline, superior fronto-occipital fasciculus, ipsilateral cerebral penduncles, sagittal stratum, corona radiata and external capsules.

The following publications were reviewed to inform the above ROI strategy: Brown et al. (2014), Catani et al. (2002), Catani, Jones, and Ffytche (2005), Chen et al. (2015), Eluvathingal et al. (2007), Kamali et al. (2014), Martino et al. (2013), Nucifora et al. (2005), Parker et al. (2005), Bain et al. (2019), and Talozzi et al. (2018)

Corticospinal tract

Corticospinal tract tracography strategy differed between the atlas creation and general tractography applied to new subjects.

Seed (atlas) For the orientation atlas, Freesurfer cortical parcellations were used to obtain more complete coverage of the motor cortex via the following process:

1. Seed in precentral gyrus and output successful seed location

2. Generate binary mask from successful seed locations, subtract from precentral gyrus mask to create seed mask

3. Re-run tractography with second seed mask to cover rest of precentral gyrus

Seed (general) Posterior limb of internal capsule, drawn on 3 consecutive axial slices

Include Posterior limb on internal capsule (if not used for seed), cerebral pendumcles, CST in mid-pons

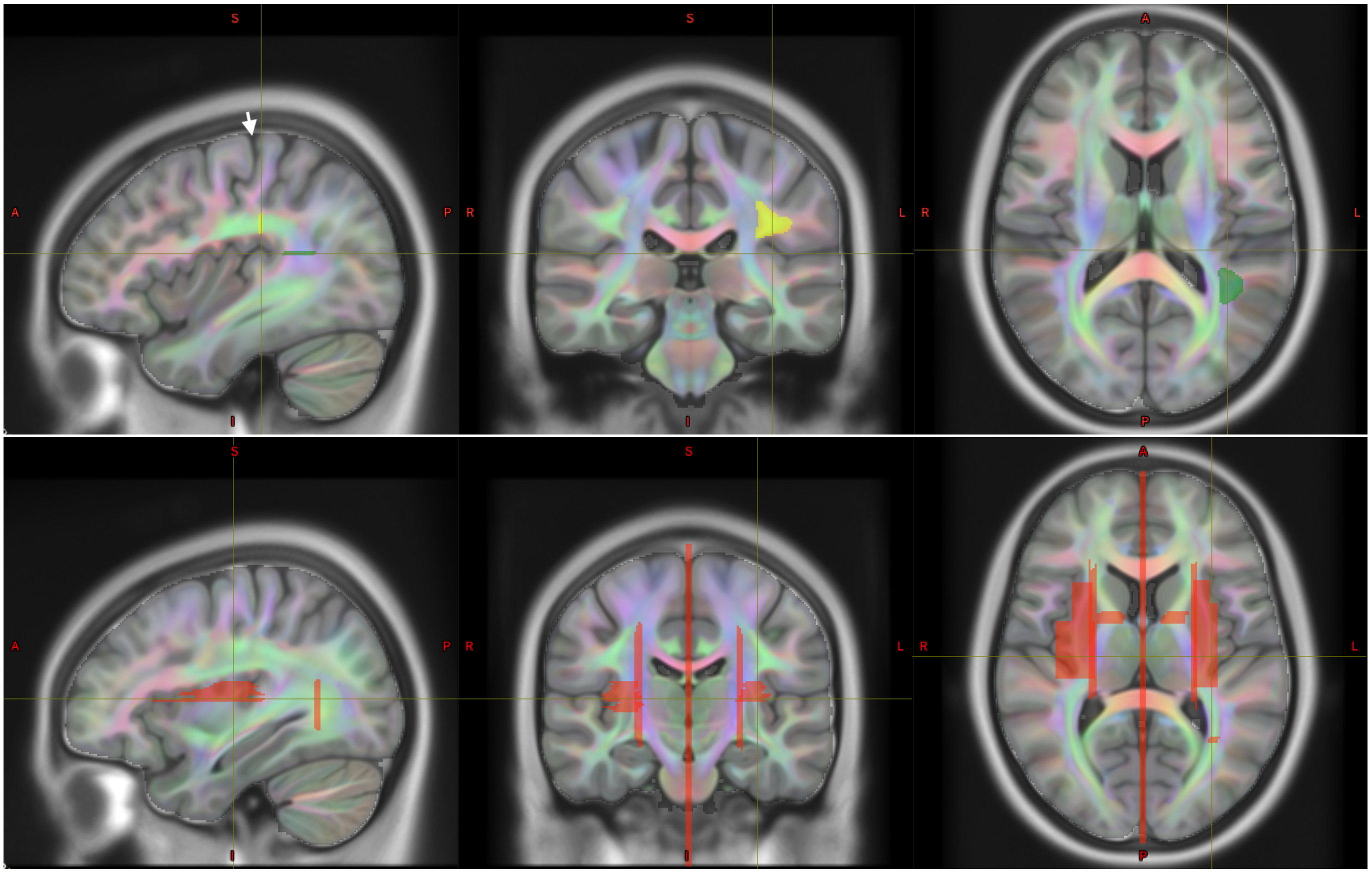

Figure D.3 Seed (yellow), inclusion (green) and exclusion (red) regions of interest for the arcuate fasciculus. Arrow indicates central sulcus, landmark for seed ROI.

Exclude Cerebellar peduncles (drawn on coronal slice), medial lemniscus (drawn on axial slice), midline, superior fronto-occipital fasciculus,

The following publications were reviewed to inform the above ROI strategy: Ciccarelli et al. (2006), Han et al. (2010), Hattingen et al. (2009), Niu et al. (2016), Radmanesh et al. (2015), Reich et al. (2006), Rosenstock et al. (2017), Szmuda et al. (2021), and Vargas et al. (2013)

Inferior fronto-occipital fasciculus

Seed Temporal stem, between anterior tip if Meyer's loop and descending portion of the uncinate fasciculus

Include (atlas) Posterior: inferior, middle, and superior occipital gyri and middle and superior occipital sulci (Freesurfer (v4.5) Destrieux atlas(Destrieux et al., 2010) (2009 version) parcellation labels 1{1,2}1{02,19,20,58,59}). Anterior: frontal pole, middle and inferior frontal gyri and sulci, orbital gyrus and sulci (Freesurfer labels 1{1,2}1{01,05,15,54,12,13,14,53,63,24,65})

Include (general) Frontal lobe coronal slice, anterior to genu of the corpus callosum

Exclude Coronal slice on frontal lobe at the level of the central sulcus, coronal slice on tip of anterior temporal lobe

The following publications reviewed to inform the above ROI strategy: Martino et al. (2010), Sarubbo et al. (2013), Hau et al. (2016), Catani and Schotten (2008), Wakana et al. (2007), and Wu et al. (2016)

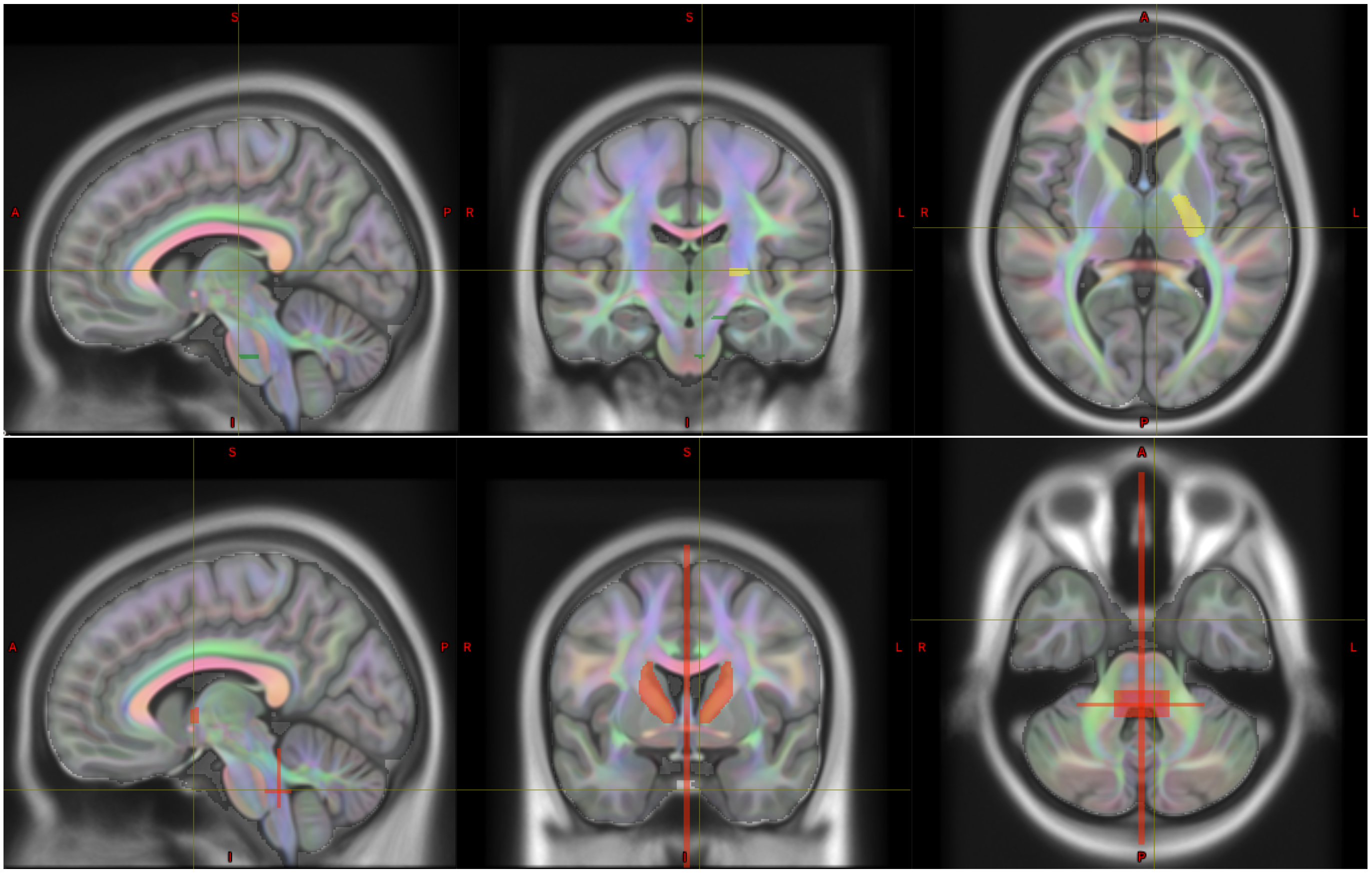

Figure D.4 Seed (yellow), inclusion (green) and exclusion (red) regions of interest for the corticospinal tract

Optic radiation

Seed Lateral geniculate nucleus (LGN; drawn on axial planes)

Include Sagittal stratum (drawn on coronal plane)

Exclude Coronal slice anterior of and axial slice inferior of most anterior point of lateral ventricles, axial slice at level of superior reach lateral ventricles, splenium of corpus callosum, fornix

The following publications reviewed to inform the above ROI strategy: Yogarajah et al. (2009), Hofer, Karaus, and Frahm (2010), and Dayan, Kreutzer, and Clark (2015)

D.2 Tracking parameters

Default parameters as documented for the `tckgen` command of MRtrix3 (release version 3.0.3, available at https://mrtrix.readthedocs.io/en/3.0.3/reference/commands/tckgen.html) (including `-select 5000 -algorithm iFOD2`) were used for all tractography. In addition, the parameter `-seed_unidirectional` was included for optic radiation reconstructions, to ensure streamlines are propagated from a single direction out of the LGN.

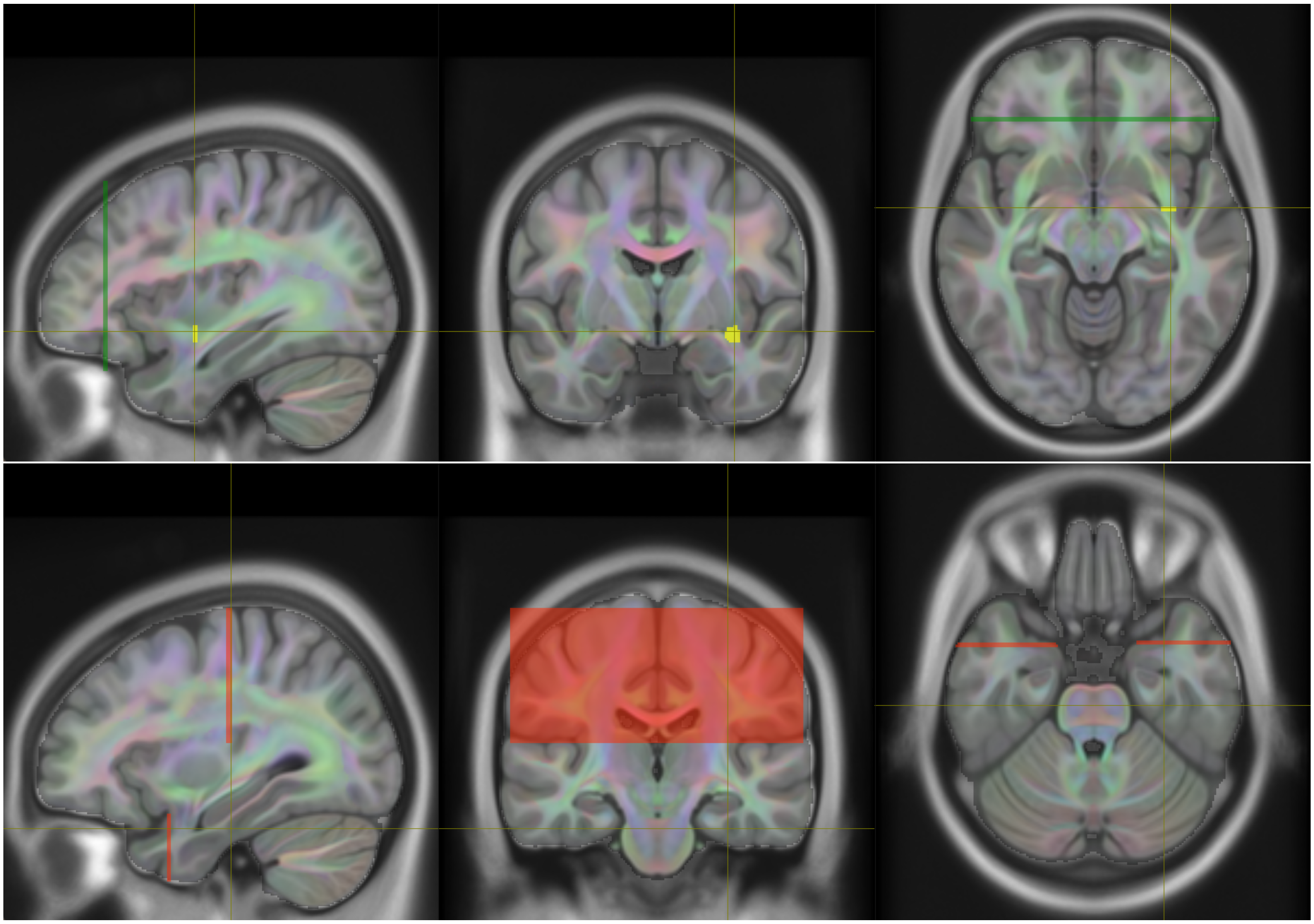

Figure D.5 Seed (yellow), inclusion (green) and exclusion (red) regions of interest for the inferior fronto-occipital fasciculus

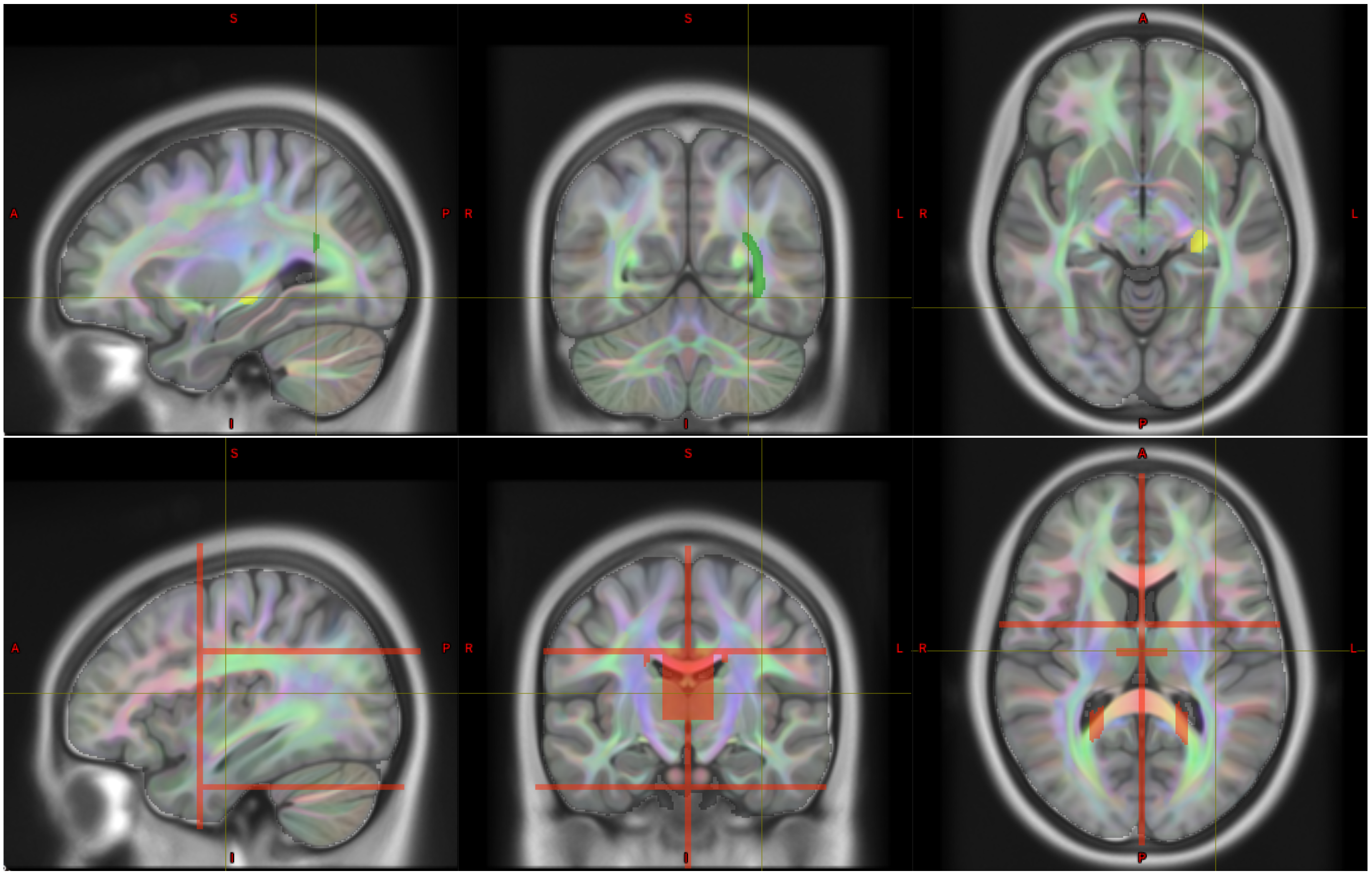

Figure D.6 Seed (yellow), inclusion (green) and exclusion (red) regions of interest for the optic radiation

E Supplementary figures

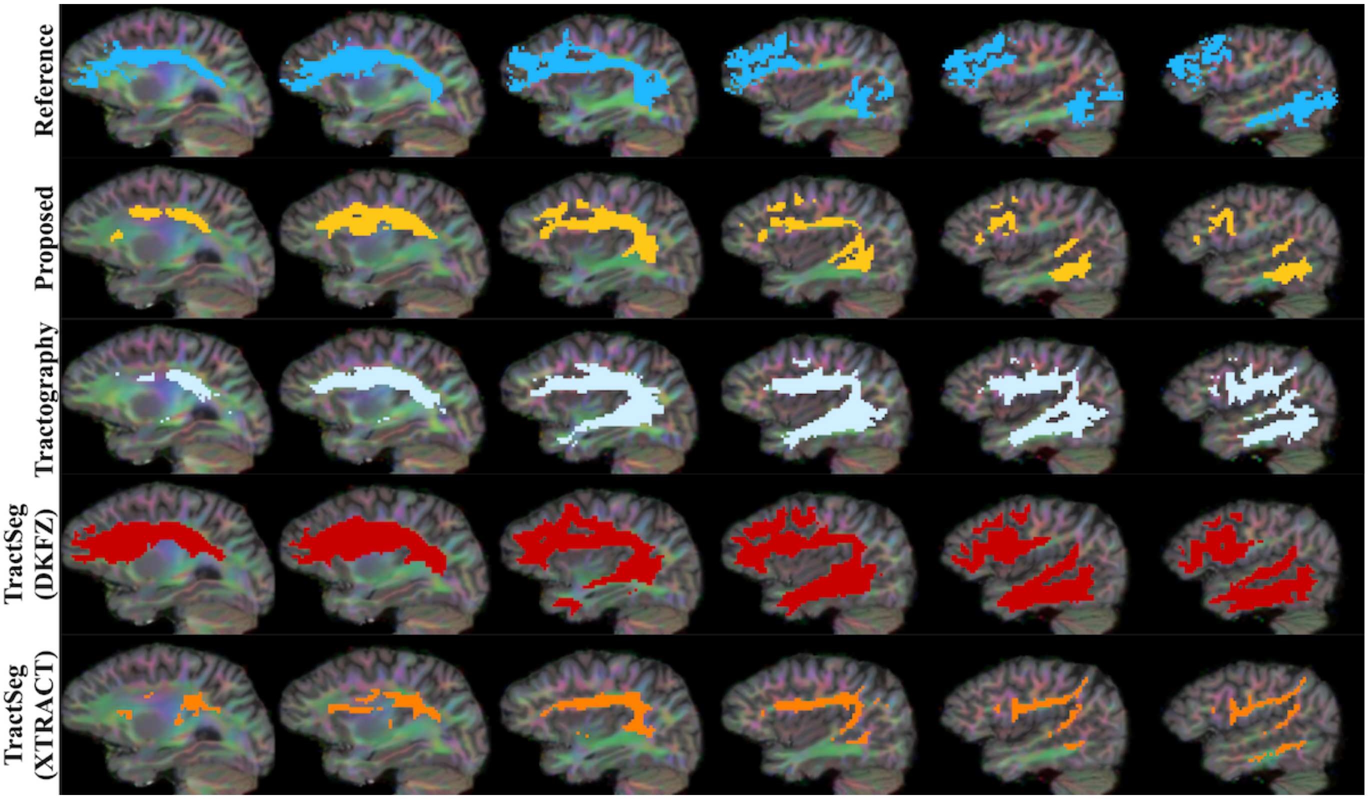

Figure E.7 TractoInferno subject 1099: Right arcuate fasciculus. Intensity thresholds are as described in Table **??**

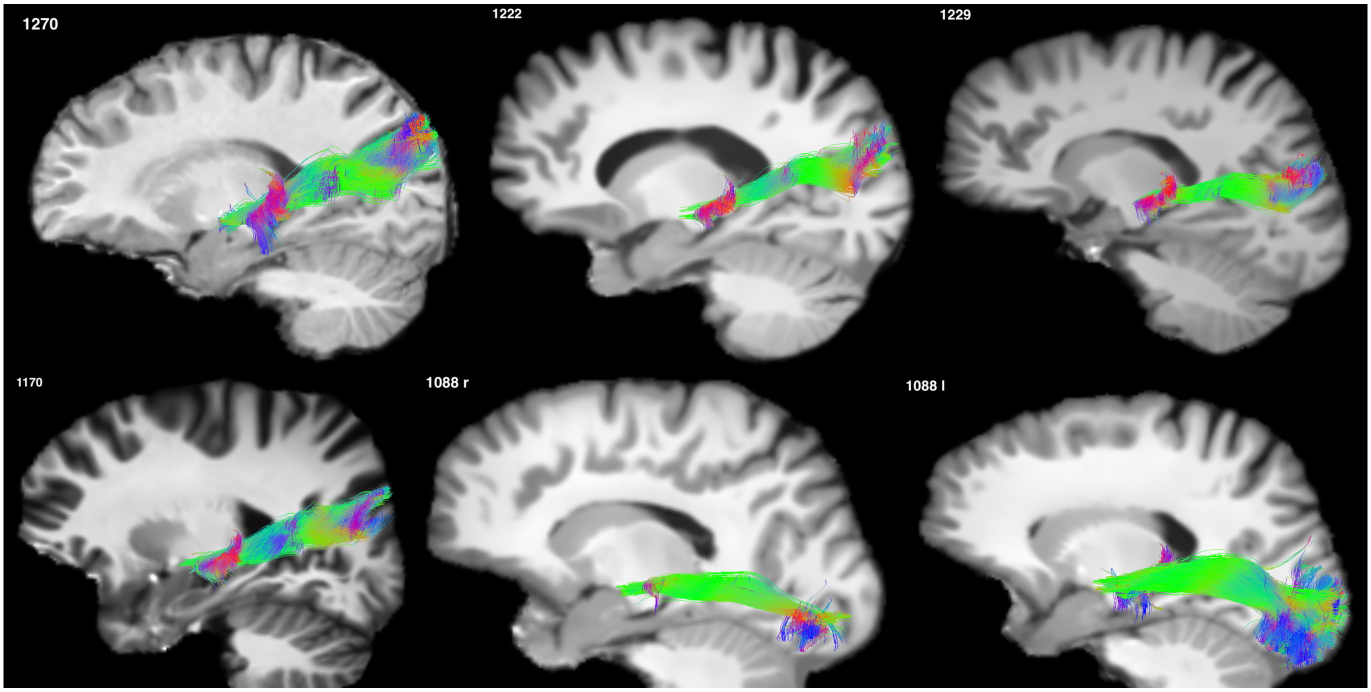

(a) Examples of incomplete optic radiation bundles, reaching only a portion of the visual cortex, in the TractoInferno dataset. Bundles such as these are the cause of the outliers seen in Fig **??**. For one subject, contralateral bundle is shown for comparison

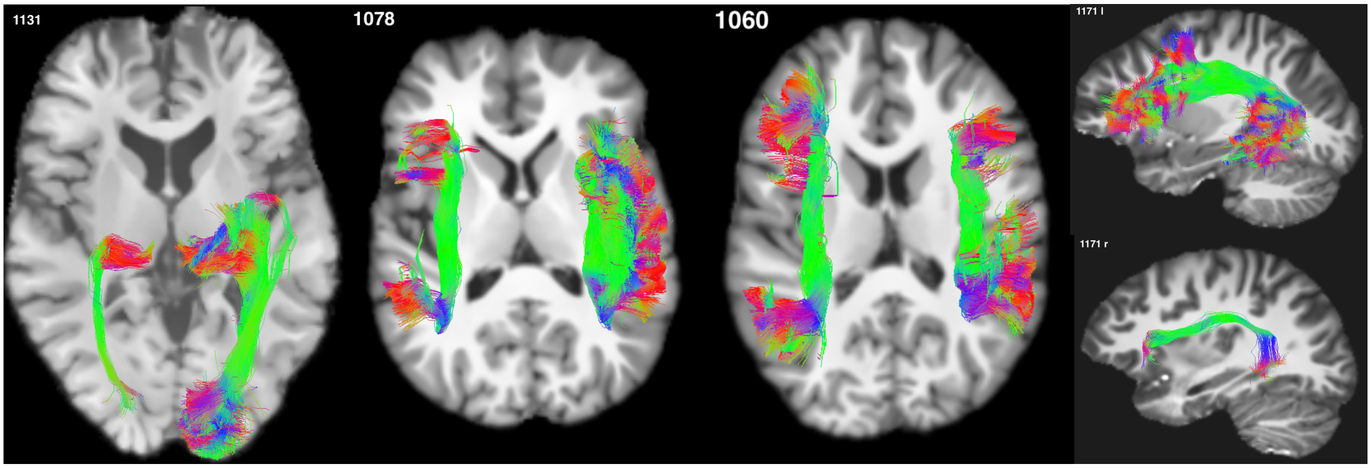

(b) Examples of strongly asymmetric bundles in the TractoInferno dataset.

Figure E.8

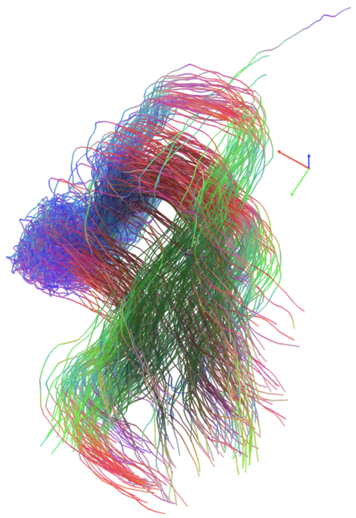

Figure E.9 Examples of orientational false positive streamlines, isolated from a corticospinal tract bundle. View is from a superior vantage. Ascending streamlines following the CST abruptly turn along an anterior-posterior direction to follow the course of the intersecting superior longitudinal fasciculus, before again turning back towards the motor cortex.

F All pairwise comparisons data

Table 1 All pairwise comparisons for HCP dataset. Bundle distances are in mm, all other metrics are dimensionless. AF=arcuate fasciculus, CST=corticospinal tract, IFOF=inferior fronto-occipital fasciculus, OR=optic radiation, TF=tractfinder (proposed), TGR=reference tractography (TractoInferno streamlines), TG=in-house tractography, TSD=TractSeg (DKFZ), TSX=TractSeg (XTRACT), AT=atlas.

Dice similarity coefficient		AT	TF	TG	TSD	TSX	density correlation	
			0.77±0.03	0.49±0.07	0.71±0.04	0.55±0.06	AT	AF
			0.77±0.03	0.49±0.04	0.74±0.05	0.65±0.06	AT	CST
			0.80±0.03	0.49±0.05	0.75±0.06	0.63±0.06	AT	IFO
			0.79±0.03	0.51±0.06	0.68±0.06	0.57±0.08	AT	OR
TF	AF	0.60±0.07		0.66±0.09	0.62±0.03	0.64±0.05	TF	AF
TF	CST	0.67±0.05		0.63±0.04	0.75±0.03	0.78±0.05	TF	CST
TF	IFO	0.68±0.06		0.55±0.06	0.64±0.03	0.70±0.04	TF	IFO
TF	OR	0.68±0.06		0.60±0.06	0.64±0.04	0.67±0.06	TF	OR
TG	AF	0.51±0.06	0.46±0.07		0.43±0.05	0.62±0.06	TG	AF
TG	CST	0.63±0.05	0.69±0.04		0.52±0.03	0.59±0.03	TG	CST
TG	IFO	0.54±0.04	0.51±0.04		0.44±0.03	0.61±0.03	TG	IFO
TG	OR	0.63±0.05	0.61±0.05		0.51±0.03	0.63±0.04	TG	OR
TSD	AF	0.53±0.04	0.39±0.07	0.61±0.06		0.57±0.02	TSD	AF
TSD	CST	0.57±0.05	0.67±0.03	0.62±0.02		0.69±0.02	TSD	CST
TSD	IFO	0.59±0.05	0.50±0.05	0.56±0.03		0.65±0.01	TSD	IFO
TSD	OR	0.54±0.07	0.53±0.05	0.64±0.04		0.66±0.02	TSD	OR
TSX	AF	0.34±0.04	0.47±0.05	0.39±0.03	0.29±0.02			
TSX	CST	0.39±0.05	0.55±0.04	0.51±0.04	0.51±0.02			
TSX	IFO	0.41±0.05	0.53±0.04	0.46±0.03	0.39±0.02			
TSX	OR	0.34±0.05	0.46±0.05	0.41±0.03	0.45±0.02			

bundle distance		AT	TF	TG	TSD	TSX	signed bundle distance	
			2.94±0.42	-2.57±0.98	-4.80±0.98	3.66±0.57	AT	AF
			2.63±0.38	2.28±1.39	2.73±0.46	7.25±0.75	AT	CST
			2.22±0.51	-2.17±0.80	-2.99±0.69	3.78±0.53	AT	IFO
			2.40±0.47	0.56±0.91	1.97±1.07	4.10±0.64	AT	OR
TF	AF	3.25±0.30		5.51±0.95	6.40±0.93	-0.60±1.22	TF	AF
TF	CST	3.13±0.24		0.93±1.19	-0.14±0.76	-5.56±0.77	TF	CST
TF	IFO	2.99±0.21		4.12±0.78	4.13±0.68	-2.09±0.94	TF	IFO
TF	OR	2.98±0.25		1.79±0.86	0.58±1.09	-2.22±0.78	TF	OR
TG	AF	5.45±0.90	6.20±0.94		-2.68±1.19	5.40±0.49	TG	AF
TG	CST	4.78±1.04	4.03±0.66		1.38±0.96	4.69±0.71	TG	CST
TG	IFO	5.49±0.47	5.52±0.51		0.03±1.12	-4.71±0.38	TG	IFO
TG	OR	3.74±0.50	3.77±0.44		1.27±0.91	4.26±0.58	TG	OR
TSD	AF	5.91±0.78	6.47±0.91	5.41±1.05		7.22±0.66	TSD	AF
TSD	CST	4.47±0.44	4.32±0.32	5.16±0.52		4.77±0.56	TSD	CST
TSD	IFO	5.14±0.79	5.04±0.75	5.96±0.54		-5.31±0.35	TSD	IFO
TSD	OR	5.52±0.50	5.14±0.39	5.26±0.47		3.26±0.80	TSD	OR
TSX	AF	5.38±0.60	4.91±0.57	5.89±0.53	7.67±0.76			
TSX	CST	7.64±0.85	6.50±0.74	5.02±0.70	6.37±0.51			
TSX	IFO	5.14±0.38	4.58±0.32	4.93±0.36	5.97±0.28			
TSX	OR	5.52±0.74	4.86±0.53	5.90±0.47	5.52±0.33			

Table 2 All pairwise comparisons for clinical dataset. Bundle distances are in *mm*, all other metrics are dimensionless. AF=arcuate fasciculus, CST=corticospinal tract, IFOF=inferior fronto-occipital fasciculus, OR=optic radiation, TF=tractfinder (proposed), TGR=reference tractography (TractoInferno streamlines), TG=in-house tractography, TSD=TractSeg (DKFZ), TSX=TractSeg (XTRACT), AT=atlas.

Dice similarity coefficient (lower) / density correlation (upper)		AT	TF	TG	TSD	TSX		
			0.79±0.05	0.44±0.08	0.72±0.04	0.58±0.06	AT	AF
			0.77±0.04	0.42±0.05	0.74±0.07	0.63±0.06	AT	CST
			0.80±0.05	0.43±0.07	0.72±0.07	0.63±0.06	AT	IFO
			0.78±0.05	0.44±0.09	0.70±0.05	0.60±0.06	AT	OR
TF	AF	0.65±0.09		0.63±0.10	0.63±0.04	0.68±0.05	TF	AF
TF	CST	0.70±0.06		0.61±0.06	0.76±0.03	0.78±0.05	TF	CST
TF	IFO	0.68±0.09		0.57±0.10	0.62±0.05	0.74±0.04	TF	IFO
TF	OR	0.66±0.09		0.58±0.11	0.62±0.05	0.70±0.04	TF	OR
TG	AF	0.49±0.10	0.54±0.10		0.35±0.06	0.56±0.11	TG	AF
TG	CST	0.54±0.07	0.65±0.06		0.45±0.05	0.55±0.07	TG	CST
TG	IFO	0.50±0.06	0.55±0.06		0.38±0.05	0.57±0.11	TG	IFO
TG	OR	0.55±0.09	0.62±0.06		0.44±0.07	0.58±0.08	TG	OR
TSD	AF	0.53±0.04	0.45±0.08	0.49±0.09		0.58±0.04	TSD	AF
TSD	CST	0.51±0.11	0.63±0.09	0.55±0.08		0.68±0.03	TSD	CST
TSD	IFO	0.54±0.07	0.48±0.07	0.51±0.07		0.64±0.04	TSD	IFO
TSD	OR	0.51±0.09	0.49±0.07	0.55±0.09		0.67±0.05	TSD	OR
TSX	AF	0.27±0.10	0.39±0.13	0.32±0.12	0.21±0.06			
TSX	CST	0.29±0.10	0.42±0.14	0.44±0.14	0.43±0.08			
TSX	IFO	0.35±0.10	0.48±0.10	0.43±0.12	0.33±0.06			
TSX	OR	0.31±0.08	0.45±0.10	0.44±0.10	0.44±0.05			

bundle distance (lower) / signed bundle distance (upper)		AT	TF	TG	TSD	TSX		
			2.72±0.84	0.79±1.88	-3.66±2.86	6.56±3.65	AT	AF
			2.37±0.53	2.38±1.52	3.66±1.82	8.76±2.01	AT	CST
			2.29±0.80	-0.68±1.65	-2.15±1.74	5.55±1.97	AT	IFO
			2.66±0.62	2.18±1.36	2.17±1.70	5.40±1.98	AT	OR
TF	AF	3.20±0.59		2.40±1.36	5.44±1.61	-4.21±3.86	TF	AF
TF	CST	3.01±0.36		-0.06±1.50	-1.60±1.96	-7.19±1.89	TF	CST
TF	IFO	2.96±0.38		3.21±1.33	4.09±1.10	-3.60±1.91	TF	IFO
TF	OR	3.08±0.36		0.46±1.21	0.44±1.97	-3.31±2.02	TF	OR
TG	AF	5.25±1.57	5.18±1.49		-4.66±2.64	6.51±3.69	TG	AF
TG	CST	5.07±0.93	4.64±0.84		2.19±2.81	6.96±3.37	TG	CST
TG	IFO	5.25±0.87	5.31±0.92		-0.81±1.99	-5.93±2.03	TG	IFO
TG	OR	4.03±0.61	3.68±0.48		-0.36±2.47	4.07±2.75	TG	OR
TSD	AF	5.77±1.34	5.87±1.07	6.24±1.92		9.16±2.42	TSD	AF
TSD	CST	4.96±1.58	4.33±1.18	6.16±1.50		5.74±1.32	TSD	CST
TSD	IFO	5.01±0.78	5.24±0.81	5.91±1.22		-6.92±1.78	TSD	IFO
TSD	OR	5.44±0.67	5.33±0.49	5.62±0.80		4.61±1.45	TSD	OR
TSX	AF	7.33±3.31	6.07±2.83	7.66±2.75	9.34±2.37			
TSX	CST	8.97±1.93	7.55±1.74	7.21±3.28	6.68±1.20			
TSX	IFO	6.18±1.70	4.91±1.19	6.27±1.91	7.19±1.71			
TSX	OR	6.19±1.67	5.12±1.15	6.07±1.57	5.98±1.13			

Table 3 All pairwise comparisons for TractoInferno dataset. Bundle distances are in *mm*, all other metrics are dimensionless. AF=arcuate fasciculus, CST=corticospinal tract, IFOF=inferior fronto-occipital fasciculus, OR=optic radiation, TF=tractfinder (proposed), TGR=reference tractography (TractoInferno streamlines), TG=in-house tractography, TSD=TractSeg (DKFZ), TSX=TractSeg (XTRACT), AT=atlas.

Dice similarity coefficient

		AT	TF	TG	TGR	TSD
TF	AF	0.66±0.04				
	CST	0.68±0.03				
	IFO	0.68±0.04				
	OR	0.69±0.04				
TG	AF	0.54±0.05	0.52±0.05			
	CST	0.56±0.04	0.68±0.04			
	IFO	0.49±0.03	0.51±0.03			
	OR	0.64±0.04	0.65±0.03			
TGR	AF	0.49±0.07	0.45±0.07	0.49±0.09		
	CST	0.58±0.04	0.48±0.04	0.48±0.04		
	IFO	0.51±0.03	0.42±0.04	0.48±0.04		
	OR	0.48±0.09	0.44±0.05	0.52±0.08		
TSD	AF	0.57±0.03	0.47±0.04	0.60±0.05	0.57±0.10	
	CST	0.53±0.06	0.67±0.06	0.60±0.04	0.45±0.06	
	IFO	0.61±0.04	0.52±0.04	0.48±0.03	0.66±0.04	
	OR	0.55±0.05	0.51±0.04	0.61±0.04	0.59±0.08	
TSX	AF	0.30±0.04	0.45±0.05	0.36±0.04	0.25±0.06	0.26±0.02
	CST	0.36±0.04	0.53±0.04	0.43±0.04	0.27±0.04	0.49±0.04
	IFO	0.40±0.03	0.56±0.03	0.42±0.04	0.31±0.05	0.38±0.02
	OR	0.33±0.04	0.47±0.05	0.41±0.04	0.38±0.05	0.46±0.03

density correlation

		TF	TG	TGR	TSD	TSX
AT	AF	0.80±0.02	0.50±0.05	0.35±0.07	0.74±0.02	0.55±0.05
	CST	0.76±0.03	0.44±0.04	0.41±0.04	0.73±0.05	0.66±0.05
	IFO	0.80±0.02	0.44±0.04	0.35±0.04	0.77±0.04	0.64±0.03
	OR	0.81±0.02	0.55±0.04	0.34±0.06	0.70±0.04	0.58±0.05
TF	AF		0.68±0.07	0.48±0.10	0.65±0.03	0.66±0.04
	CST		0.62±0.05	0.60±0.05	0.77±0.03	0.79±0.04
	IFO		0.54±0.06	0.50±0.06	0.66±0.02	0.76±0.02
	OR		0.69±0.04	0.45±0.08	0.63±0.03	0.69±0.04
TG	AF			0.50±0.14	0.43±0.04	0.61±0.05
	CST			0.57±0.10	0.49±0.04	0.51±0.04
	IFO			0.55±0.09	0.39±0.03	0.53±0.05
	OR			0.52±0.12	0.54±0.03	0.65±0.05
TGR	AF				0.29±0.05	0.40±0.10
	CST				0.51±0.04	0.61±0.05
	IFO				0.34±0.04	0.51±0.05
	OR				0.39±0.06	0.50±0.08
TSD	AF					0.57±0.02
	CST					0.68±0.03
	IFO					0.66±0.01
	OR					0.68±0.02

bundle distance

		AT	TF	TG	TGR	TSD
TF	AF	3.13±0.19				
	CST	3.26±0.24				
	IFO	3.17±0.29				
	OR	3.21±0.32				
TG	AF	5.06±0.86	5.54±0.89			
	CST	4.47±0.62	3.87±0.58			
	IFO	6.50±0.74	6.34±0.56			
	OR	3.42±0.39	3.56±0.33			
TGR	AF	5.33±1.14	6.17±1.36	6.25±1.32		
	CST	5.57±0.58	5.95±0.54	6.44±0.63		
	IFO	5.48±0.59	6.33±0.72	7.09±0.90		
	OR	5.30±1.04	5.47±0.71	5.20±1.01		
TSD	AF	5.27±0.76	5.91±0.87	5.07±0.85	5.20±1.69	
	CST	5.16±1.35	4.38±0.82	4.94±0.62	7.20±1.40	
	IFO	4.69±0.67	5.10±0.70	7.21±0.84	4.68±0.74	
	OR	5.59±0.39	5.44±0.30	5.35±0.49	4.97±0.96	
TSX	AF	6.17±0.75	4.92±0.54	6.35±0.82	9.03±1.63	8.41±0.84
	CST	8.32±0.72	6.87±0.80	6.81±0.99	9.43±1.27	6.58±0.72
	IFO	5.40±0.46	4.10±0.33	6.15±0.59	7.18±0.75	6.11±0.38
	OR	5.89±0.81	4.89±0.59	5.60±0.53	6.43±0.77	5.51±0.36

signed bundle distance

		TF	TG	TGR	TSD	TSX
AT	AF	2.83±0.30	-1.65±1.13	1.97±2.89	-4.07±0.99	5.10±0.88
	CST	2.78±0.32	1.81±0.63	4.20±0.99	3.69±1.44	8.10±0.69
	IFO	2.72±0.42	-1.09±1.34	3.75±1.27	-2.23±0.69	4.52±0.63
	OR	2.89±0.41	0.93±0.78	-0.77±2.72	2.31±1.26	4.74±0.78
TF	AF		4.59±0.94	4.62±2.69	5.82±0.89	-2.57±1.05
	CST		1.24±0.73	5.59±0.69	-1.07±1.30	-6.36±0.85
	IFO		4.31±1.02	5.71±1.03	4.35±0.78	-2.27±0.79
	OR		1.95±0.81	1.86±2.66	0.32±1.33	-2.60±0.71
TG	AF			0.16±3.04	-2.60±0.97	6.03±0.86
	CST			5.49±0.78	2.29±1.04	6.07±0.91
	IFO			3.23±1.82	-1.15±1.50	-5.67±0.54
	OR			-0.22±2.68	1.27±1.24	4.21±0.68
TGR	AF				-2.46±2.95	7.68±2.60
	CST				7.00±1.43	9.43±1.27
	IFO				2.69±1.72	6.86±0.92
	OR				1.68±2.68	4.18±2.53
TSD	AF					8.15±0.83
	CST					4.82±0.95
	IFO					-5.61±0.42
	OR					3.55±1.01

References

Bain, Jonathan S., Jason D. Yeatman, Roey Schurr, Ariel Rokem, and Aviv A. Mezer (Sept. 20, 2019). "Evaluating arcuate fasciculus laterality measurements across dataset and tractography pipelines". In: *Human Brain Mapping* 40.13, pp. 3695–3711. DOI: 10.1002/hbm.24626. pmid: 31106944.

Brown, Erik C., Jeong Won Jeong, Otto Muzik, Robert Rothermel, Naoyuki Matsuzaki, Csaba Juhász, Sandeep Sood, and Eishi Asano (May 1, 2014). "Evaluating the arcuate fasciculus with combined diffusion-weighted MRI tractography and electrocorticography". In: *Human Brain Mapping* 35.5, pp. 2333–2347. DOI: 10.1002/HBM.22331. pmid: 23982893.

Catani, Marco, Robert J. Howard, Sinisa Pajevic, and Derek K. Jones (Sept. 1, 2002). "Virtual in Vivo Interactive Dissection of White Matter Fasciculi in the Human Brain". In: *NeuroImage* 17.1, pp. 77–94. DOI: 10.1006/NIMG.2002.1136. pmid: 12482069.

Catani, Marco, Derek K. Jones, and Dominic H. Ffytche (Jan. 1, 2005). "Perisylvian language networks of the human brain". In: *Annals of Neurology* 57.1, pp. 8–16. DOI: 10.1002/ANA.20319. pmid: 15597383.

Catani, Marco and Michel Thiebaut de Schotten (2008). "A diffusion tensor imaging tractography atlas for virtual in vivo dissections". In: *Cortex* 44.8, pp. 1105–1132. DOI: 10.1016/j.cortex.2008.05.004.

Chamberland, Maxime, Chantal M.W. Tax, and Derek K. Jones (2018). "Meyer's loop tractography for image-guided surgery depends on imaging protocol and hardware". In: *NeuroImage: Clinical* 20 (February), pp. 458–465. DOI: 10.1016/j.nicl.2018.08.021. pmid: 30128284.

Chen, Zhenrui, Yanmei Tie, Olutayo Olubiyi, Laura Rigolo, Alireza Mehrtash, Isaiah Norton, Ofer Pasternak, Yogesh Rathi, Alexandra J. Golby, and Lauren J. O'Donnell (Jan. 1, 2015). "Reconstruction of the arcuate fasciculus for surgical planning in the setting of peritumoral edema using two-tensor unscented Kalman filter tractography". In: *NeuroImage: Clinical* 7, pp. 815–822. DOI: 10.1016/J.NICL.2015.03.009. pmid: 26082890.

Ciccarelli, O., T. E. Behrens, D. R. Altmann, R. W. Orrell, R. S. Howard, H. Johansen-Berg, D. H. Miller, P. M. Matthews, and A. J. Thompson (July 1, 2006). "Probabilistic diffusion tractography: A potential tool to assess the rate of disease progression in amyotrophic lateral sclerosis". In: *Brain* 129.7, pp. 1859–1871. DOI: 10.1093/brain/awl100. pmid: 16672290.

Dayan, Michael, Sylvia Kreutzer, and Chris A. Clark (Apr. 1, 2015). "Tractography of the optic radiation: a repeatability and reproducibility study". In: *NMR in Biomedicine* 28.4, pp. 423–431. DOI: 10.1002/nbm.3266. pmid: 25703088.

Desikan, Rahul S, Florent Ségonne, Bruce Fischl, Brian T Quinn, Bradford C Dickerson, Deborah Blacker, Randy L Buckner, Anders M Dale, R Paul Maguire, Bradley T Hyman, Marilyn S Albert, and Ronald J Killiany (2006). "An automated labeling system for subdividing the human cerebral cortex on MRI scans into gyral based regions of interest". In: *NeuroImage* 31.3, pp. 968–980. DOI: DOI:10.1016/j.neuroimage.2006.01.021.

Destrieux, Christophe, Bruce Fischl, Anders Dale, and Eric Halgren (Oct. 15, 2010). "Automatic parcellation of human cortical gyri and sulci using standard anatomical nomenclature". In: *NeuroImage* 53.1, pp. 1–15. DOI: 10.1016/j.neuroimage.2010.06.010.

Eluvathingal, Thomas J., Khader M. Hasan, Larry Kramer, Jack M. Fletcher, and Linda Ewing-Cobbs (Dec. 1, 2007). "Quantitative Diffusion Tensor Tractography of Association and Projection Fibers in Normally Developing Children and Adolescents". In: *Cerebral Cortex* 17.12, pp. 2760–2768. DOI: 10.1093/CERCOR/BHM003. pmid: 17307759.

Fischl, Bruce, David H. Salat, Evelina Busa, Marilyn Albert, Megan Dieterich, Christian Haselgrove, Andre van der Kouwe, Ron Killiany, David Kennedy, Shuna Klaveness, Albert Montillo, Nikos Makris, Bruce Rosen, and Anders M. Dale (Jan. 31, 2002). "Whole Brain Segmentation: Automated Labeling of Neuroanatomical Structures in the Human Brain". In: *Neuron* 33.3, pp. 341–355. DOI: 10.1016/S0896-6273(02)00569-X.

Forkel, Stephanie J., Michel Thiebaut de Schotten, Jamie M. Kawadler, Flavio Dell'Acqua, Adrian Danek, and Marco Catani (July 1, 2014). "The anatomy of fronto-occipital connections from early blunt dissections to contemporary tractography". In: *Cortex*. The clinical neuroanatomy of the occipital lobes 56, pp. 73–84. DOI: 10.1016/j.cortex.2012.09.005.

Han, Bong Soo, Ji Heon Hong, Cheolpyo Hong, Sang Seok Yeo, Dong hoon Lee, Hee Kyung Cho, and Sung Ho Jang (Apr. 22, 2010). "Location of the corticospinal tract at the corona radiata in human brain". In: *Brain Research* 1326, pp. 75–80. doi: 10.1016/j.brainres.2010.02.050. pmid: 20219443.

Hattingen, Elke, Julian Rathert, Alina Jurcoane, Stefan Weidauer, Andrea Szelényi, George Ogrezeanu, Volker Seifert, Friedhelm E. Zanella, and Thomas Gasser (May 13, 2009). "A standardised evaluation of pre-surgical imaging of the corticospinal tract: Where to place the seed ROI". In: *Neurosurgical Review* 32.4, pp. 445–456. doi: 10.1007/s10143-009-0197-1. pmid: 19437053.

Hau, Janice, Silvio Sarubbo, Guy Perchey, Fabrice Crivello, Laure Zago, Emmanuel Mellet, Gaël Jobard, Marc Joliot, Bernard M. Mazoyer, Nathalie Tzourio-Mazoyer, and Laurent Petit (2016). "Cortical Terminations of the Inferior Fronto-Occipital and Uncinate Fasciculi: Anatomical Stem-Based Virtual Dissection". In: *Frontiers in Neuroanatomy* 10 (MAY), p. 58. doi: 10.3389/FNANA.2016.00058/BIBTEX. pmid: 27252628.

Hofer, Sabine, Alexander Karaus, and Jens Frahm (Apr. 13, 2010). "Reconstruction and dissection of the entire human visual pathway using diffusion tensor MRI". In: *Frontiers in Neuroanatomy* 4 (APRIL), pp. 1–7. doi: 10.3389/fnana.2010.00015.

Kamali, Arash, Adam E. Flanders, Joshua Brody, Jill V. Hunter, and Khader M. Hasan (Jan. 4, 2014). "Tracing superior longitudinal fasciculus connectivity in the human brain using high resolution diffusion tensor tractography". In: *Brain Structure and Function* 219.1, pp. 269–281. doi: 10.1007/S00429-012-0498-Y/FIGURES/5. pmid: 23288254.

Kumar, A., C. Juhasz, E. Asano, S.K. Sundaram, M.I. Makki, D.C. Chugani, and H.T. Chugani (Nov. 2009). "Diffusion Tensor Imaging Study of the Cortical Origin and Course of the Corticospinal Tract in Healthy Children". In: *American Journal of Neuroradiology* 30.10, pp. 1963–1970. doi: 10.3174/ajnr.A1742. pmid: 19661173.

Lilja, Ylva and Daniel T Nilsson (2015). "Strengths and limitations of tractography methods to identify the optic radiation for epilepsy surgery." In: *Quantitative imaging in medicine and surgery* 5.2, pp. 288–28899. doi: 10.3978/j.issn.2223-4292.2015.01.08.

Martino, Juan, Christian Brogna, Santiago G. Robles, Francesco Vergani, and Hugues Duffau (May 1, 2010). "Anatomic dissection of the inferior fronto-occipital fasciculus revisited in the lights of brain stimulation data". In: *Cortex* 46.5, pp. 691–699. doi: 10.1016/j.cortex.2009.07.015.

Martino, Juan, Philip C. De Witt Hamer, Mitchel S. Berger, Michael T. Lawton, Christine M. Arnold, Enrique Marco De Lucas, and Hugues Duffau (Jan. 16, 2013). "Analysis of the subcomponents and cortical terminations of the perisylvian superior longitudinal fasciculus: A fiber dissection and DTI tractography study". In: *Brain Structure and Function* 218.1, pp. 105–121. doi: 10.1007/S00429-012-0386-5/FIGURES/8. pmid: 22422148.

Niu, Chen, Xin Liu, Yong Yang, Kun Zhang, Zhigang Min, Maode Wang, Wenfei Li, Liping Guo, Pan Lin, and Ming Zhang (2016). "Assessing region of interest schemes for the corticospinal tract in patients with brain tumors". In: *Medicine (United States)* 95.12. doi: 10.1097/MD.0000000000003189.

Nucifora, Paolo G.P., Ragini Verma, Elias R. Melhem, Raquel E. Gur, and Ruben C. Gur (2005). "Leftward asymmetry in relative fiber density of the arcuate fasciculus". In: *NeuroReport* 16.8, pp. 791–794. doi: 10.1097/00001756-200505310-00002. pmid: 15891571.

Parker, Geoffrey J.M., Simona Luzzi, Daniel C. Alexander, Claudia A.M. Wheeler-Kingshott, Olga Ciccarelli, and Matthew A. Lambon Ralph (Feb. 1, 2005). "Lateralization of ventral and dorsal auditory-language pathways in the human brain". In: *NeuroImage* 24.3, pp. 656–666. doi: 10.1016/J.NEUROIMAGE.2004.08.047. pmid: 15652301.

Radmanesh, Alireza, Amir A. Zamani, Stephen Whalen, Yanmei Tie, Ralph O. Suarez, and Alexandra J. Golby (Feb. 1, 2015). "Comparison of seeding methods for visualization of the corticospinal tracts using single tensor tractography". In: *Clinical Neurology and Neurosurgery* 129, pp. 44–49. doi: 10.1016/j.clineuro.2014.11.021. pmid: 25532134.

Reich, Daniel S, S A Smith, C K Jones, K M Zackowski, P C Van Zijl, P A Calabresi, and S Mori (2006). "Quantitative characterization of the corticospinal tract at 3T". In: *American Journal of Neuroradiology* 27.10, pp. 2168–2178. pmid: 17110689. url: www.ajnr.org (visited on 08/03/2021).

Rollans, Claire and Jacqueline Cummine (May 1, 2018). “One tract, two tract, old tract, new tract: A pilot study of the structural and functional differentiation of the inferior fronto-occipital fasciculus”. In: *Journal of Neurolinguistics* 46, pp. 122–137. DOI: 10.1016/j.jneuroling.2017.12.009.

Rosenstock, Tizian, Davide Giampiccolo, Heike Schneider, Sophia Jutta Runge, Ina Bährend, Peter Vajkoczy, and Thomas Picht (Jan. 1, 2017). “Specific DTI seeding and diffusivity-analysis improve the quality and prognostic value of TMS-based deterministic DTI of the pyramidal tract”. In: *NeuroImage: Clinical* 16, pp. 276–285. DOI: 10.1016/j.nicl.2017.08.010. pmid: 28840099.

Sarubbo, Silvio, Alessandro De Benedictis, Igor L. Maldonado, Gianpaolo Basso, and Hugues Duffau (Jan. 1, 2013). “Frontal terminations for the inferior fronto-occipital fascicle: anatomical dissection, DTI study and functional considerations on a multi-component bundle”. In: *Brain Structure and Function* 218.1, pp. 21–37. DOI: 10.1007/s00429-011-0372-3.

Szmuda, T., S. Kierońska, S. Ali, P. Sł oniewski, M. Pacholski, J. Dzierzanowski, A. Sabisz, and E. Szurowska (2021). “Tractography-guided surgery of brain tumours: what is the best method to outline the corticospinal tract?” In: *Folia Morphologica (Poland)* 80.1, pp. 40–46. DOI: 10.5603/FM.A2020.0016. pmid: 32073136.

Talozzi, Lia, Claudia Testa, Stefania Evangelisti, Lorenzo Cirignotta, Claudio Bianchini, Stefano Ratti, Paola Fantazzini, Caterina Tonon, David Neil Manners, and Raffaele Lodi (Dec. 1, 2018). “Along-tract analysis of the arcuate fasciculus using the Laplacian operator to evaluate different tractography methods”. In: *Magnetic Resonance Imaging* 54, pp. 183–193. DOI: 10.1016/j.mri.2018.08.013. pmid: 30165094.

Vargas, Patricia, Marie Gaudron, Romain Valabrègue, Eric Bertasi, Frédéric Humbert, Stéphane Lehéricy, Yves Samson, and Charlotte Rosso (Apr. 1, 2013). “Assessment of corticospinal tract (CST) damage in acute stroke patients: Comparison of tract-specific analysis versus segmentation of a CST template”. In: *Journal of Magnetic Resonance Imaging* 37.4, pp. 836–845. DOI: 10.1002/jmri.23870. pmid: 23086724.

Wakana, Setsu, Arvind Caprihan, Martina M. Panzenboeck, James H. Fallon, Michele Perry, Randy L. Gollub, Kegang Hua, Jiangyang Zhang, Hangyi Jiang, Prachi Dubey, Ari Blitz, Peter van Zijl, and Susumu Mori (July 1, 2007). “Reproducibility of quantitative tractography methods applied to cerebral white matter”. In: *NeuroImage* 36.3, pp. 630–644. DOI: 10.1016/j.neuroimage.2007.02.049.

Wasserthal, Jakob, Peter Neher, and Klaus Maier-Hein (Nov. 5, 2018a). *High quality white matter reference tracts.* Version 1.2.0. DOI: 10.5281/zenodo.1477956.

Wasserthal, Jakob, Peter Neher, and Klaus H. Maier-Hein (Dec. 1, 2018b). “TractSeg - Fast and accurate white matter tract segmentation”. In: *NeuroImage* 183, pp. 239–253. DOI: 10.1016/j.neuroimage.2018.07.070. pmid: 30086412.

Weiller, Cornelius, Marco Reisert, Ivo Peto, Jürgen Hennig, Nikos Makris, Michael Petrides, Michel Rijntjes, and Karl Egger (July 1, 2021). “The ventral pathway of the human brain: A continuous association tract system”. In: *NeuroImage* 234, p. 117977. DOI: 10.1016/j.neuroimage.2021.117977.

Wu, Yupeng, Dandan Sun, Yong Wang, and Yibao Wang (Sept. 23, 2016). “Subcomponents and connectivity of the inferior fronto-occipital fasciculus revealed by diffusion spectrum imaging fiber tracking”. In: *Frontiers in Neuroanatomy* 10 (SEP). DOI: 10.3389/FNANA.2016.00088/FULL.

Yogarajah, M., N. K. Focke, S. Bonelli, M. Cercignani, J. Acheson, G. J.M. Parker, D. C. Alexander, A. W. McEvoy, M. R. Symms, M. J. Koepp, and J. S. Duncan (June 2009). “Defining Meyers looptemporal lobe resections, visual field deficits and diffusion tensor tractography”. In: *Brain* 132.6, pp. 1656–1668. DOI: 10.1093/brain/awp114. pmid: 19460796.
